# Loss of cardiolipin and porins bypasses the essentiality of the sigma E cell envelope stress response in *Escherichia coli*

**DOI:** 10.1128/mbio.01613-25

**Published:** 2025-08-18

**Authors:** Zihao Yang, Emily C. A. Goodall, Bing Zhang, Von V. L Torres, Jessica L. Rooke, Karthik Pullela, Rochelle M. Da Costa, Christopher Icke, Mark A.T. Blaskovich, Simon Legood, Adam F. Cunningham, Waldemar Vollmer, Jack A. Bryant, Ian R. Henderson

**Affiliations:** 1 Institute for Molecular Bioscience, University of Queensland 85088, Brisbane, Queensland, Australia; 2 Centre for Superbug Solutions, University of Queensland, Brisbane, Queensland, Australia; 3 Institute of Microbiology and Infection, University of Birmingham 1724, Birmingham, England, United Kingdom; 4 School of Life Sciences, University of Nottingham 6123, Nottingham, England, United Kingdom; Massachusetts Institute of Technology, Cambridge, Massachusetts, USA

**Keywords:** *E. coli*, cardiolipin, phospholipid, transposon sequencing, tradis

## Abstract

The Gram-negative cell envelope is composed of an inner and outer membrane, each with different lipid compositions. The inner membrane is predominantly made up of phosphatidylglycerol, phosphatidylethanolamine, and cardiolipin, while the outer membrane contains the same lipids but has a predominantly lipopolysaccharide composition in the outer leaflet. The outer membrane forms a permeability barrier that blocks the entry of toxins and antibiotics; however, it is decorated with outer membrane proteins that allow nutrient uptake and the efflux of intracellular toxins. In *Escherichia coli,* the σ^E^ stress response is essential for viability but is upregulated when disruptions to outer membrane protein biogenesis occurs. Here, we used a high-throughput genetic screen to identify genetic interactions with the genes responsible for cardiolipin biosynthesis. We demonstrate that the σ^E^ stress response is not essential in a mutant lacking cardiolipin and the major outer membrane protein OmpC. This mutant also became more resistant to batimastat, a selective inhibitor of RseP that causes a lethal decrease in σ^E^ activity in *E. coli*. Finally, we reveal that the toxicity associated with decreased σ^E^ activity can be relieved by treatment with an inhibitor of the Sec translocon, which translocates outer membrane proteins across the inner membrane. We conclude that the loss of cardiolipin suppresses Sec activity, reducing the level of misfolded outer membrane proteins in the periplasm, thereby relieving the essential nature of the σ^E^ stress response.

**IMPORTANCE:**

The process of building the Gram-negative bacterial cell envelope is complex and requires careful coordination of many different pathways. Because of its essential role in maintaining cell viability, the cell envelope is an important target for new antibiotic treatments. The membranes that make up the cell envelope contain three major lipids, but the precise role of these lipids and how they influence the coordination of the different cell envelope pathways is not well understood. Our data indicate that when the synthesis of one of these lipids is abolished, coordination of cell envelope biosynthesis is dysregulated. Importantly, an essential regulatory mechanism for controlling the response to disruption of the cell envelope becomes non-essential. These findings provide new insight into cell envelope biogenesis that could be harnessed for developing antimicrobial strategies.

## INTRODUCTION

The bacterial envelope provides a semi-permeable membrane encompassing the cytoplasmic components of the cell (1, 2). Being in contact with the external medium, the bacterial envelope is the first essential barrier for the protection against physical, chemical, or biological stresses (3). The Gram-negative bacterial envelope consists of an inner membrane (IM) and an outer membrane (OM), surrounding an aqueous space called the periplasm containing a thin layer of peptidoglycan (4). Both membranes are primarily composed of glycerophospholipids (GPLs) and are decorated with integral membrane proteins and peripherally associated lipoproteins (2). However, the OM is distinct in that its inner leaflet predominantly contains GPLs, while the outer leaflet is largely composed of lipopolysaccharide (LPS) (2, 5). Divalent cations support strong lateral interactions between individual LPS molecules and combined with the hydrophobic packing of the LPS and GPL acyl chains they create an effective hydrophobic barrier. This barrier exhibits selective permeability, allowing the entry of essential nutrients while blocking the penetration of toxic substances such as antibiotics, detergents, and antimicrobial peptides (1).

**Fig 1:**
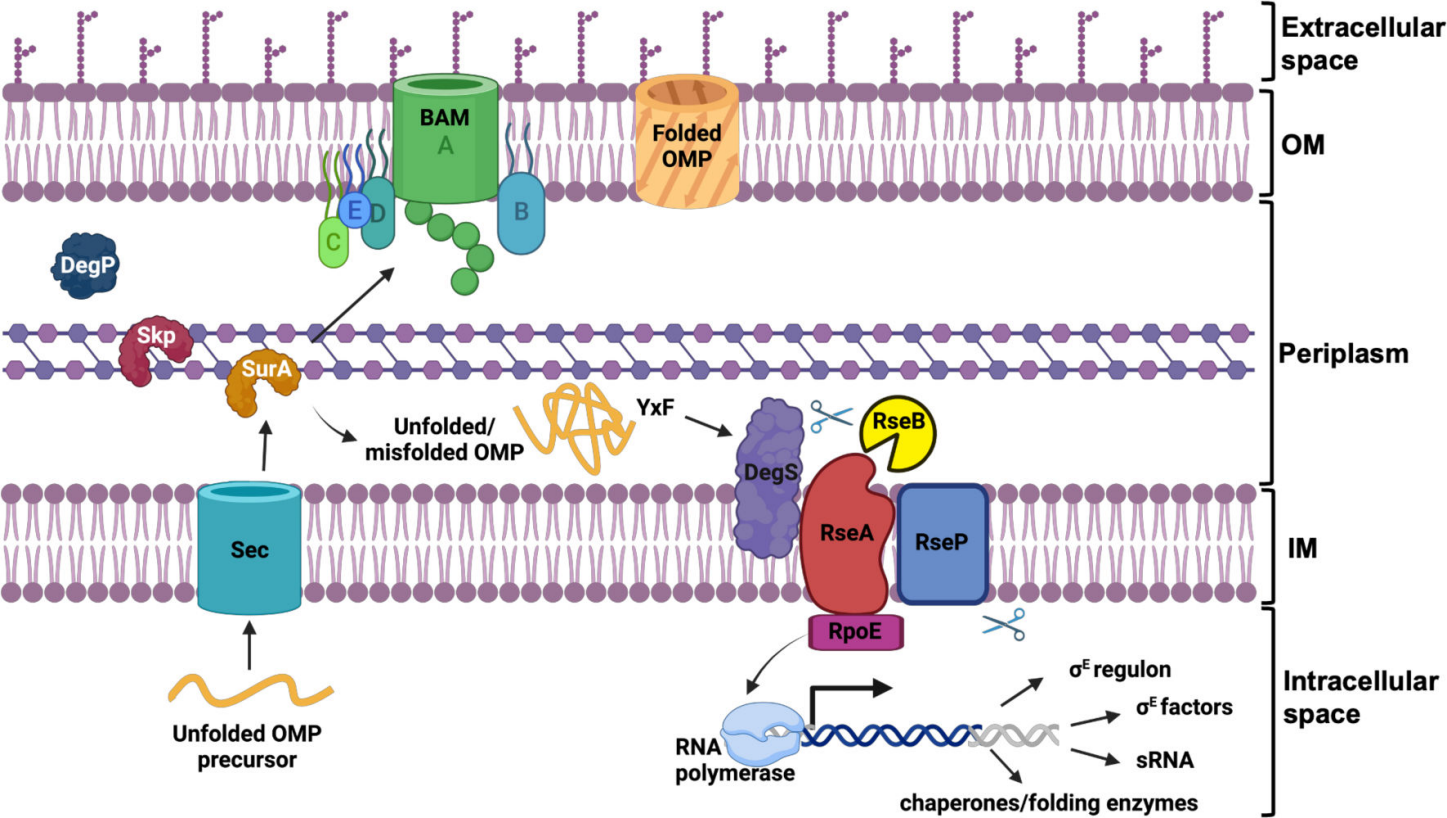
OMP biosynthesis and σ^E^ envelope stress response. OMPs are synthesized in the intracellular space, and unfolded OMPs (uOMPs) are secreted across the IM into the periplasm via the Sec translocon. uOMPs are then trafficked across the periplasm by chaperone proteins and assembled into the OM via the Bam complex for folded OMPs (fOMPs) formation. When periplasmic uOMPs accumulate, the DegS protease is activated by a substrate YxF motif, triggering cleavage of RseA. RseA is further degraded by the RseP proteases, allowing release of σ^E^ (RpoE), allowing expression from σ^E^ dependent promoters. RseB binds to the periplasmic domain of RseA and protects RseA from proteolysis. Created in BioRender.com.

To maintain the barrier function of the cell envelope, the synthesis, transport, and assembly of its constituent components must be precisely coordinated both spatially and temporally. Disruptions to this process compromise the integrity of the barrier resulting in increased sensitivity to external stresses. The response of the cell to such perturbations is primarily regulated by the alternative sigma factor, σ^E^ (3, 6). In *Escherichia coli*, σ^E^ is encoded by the essential *rpoE* gene. Under normal conditions, σ^E^ is retained at the IM in an inactive form by the anti-sigma membrane-spanning protein RseA (3, 6, 7). Upon induction of cell envelope stress, RseA is cleaved by DegS, an IM protease. This degradation renders RseA susceptible to a second protease, RseP, which releases σ^E^ from RseA (6, 7) (Fig. 1). The freed σ^E^ then interacts with RNA polymerase, initiating the expression of genes involved in the extracytoplasmic stress response (6, 7). These genes encode proteins that collectively work to restore the integrity of the cell envelope barrier (8). Like *rpoE*, the genes encoding the proteases required for σ^E^ activation, namely *degS* and *rseP*, are also essential. Remarkably, the essential nature of *rseP* can be relieved by the abolition of the major porins OmpA and OmpC, suggesting that the σ^E^ response is crucial for monitoring the production of outer membrane proteins (9).

Similar to the σ^E^ response, the synthesis of the two major membrane GPLs, phosphatidylethanolamine (PE) and phosphatidylglycerol (PG), is essential. In contrast, cardiolipin (CL), which constitutes approximately 5% of the GPLs in the cell envelope, is not essential (10). The synthesis of CL is facilitated by three distinct synthases: ClsA, ClsB, and ClsC, encoded by the genes *clsA*, *clsB*, and *clsC,* respectively (11). Although these genes are non-essential, the redundancy in the genetic pathways for CL biosynthesis suggests that CL is critical for cellular function. Indeed, CL deficiency has been associated with defects in Sec-mediated protein secretion, the proper localization of integral membrane proteins and outer membrane proteins, as well as cell division (2, 12–15). However, the exact physiological role of CL and its intersection with the σ^E^ stress response have not been elucidated.

The aim of this work was to further investigate the physiological roles of CL by comparing gene essentiality between the *E. coli* BW25113 and CL-deficient isogenic mutants using a transposon insertion site sequencing approach termed TraDIS (16). Like others, we found a synthetic lethal genetic interaction between CL production and *lpxM*, which encodes an LPS acyl transferase (17, 18). However, serendipitously, we discovered that concomitant loss of CL-production and porin production abolished the essentiality of *rpoE*. Furthermore, we demonstrate that by chemically lowering porin production, the essential nature of σ^E^ stress response is relieved. We propose a model where the σ^E^ stress response in *E. coli* is essential as CL enhances Sec-dependent protein secretion across the IM.

## RESULTS

### Creation and validation of *E. coli* BW25113 Δ*clsABC*

**Fig 2:**
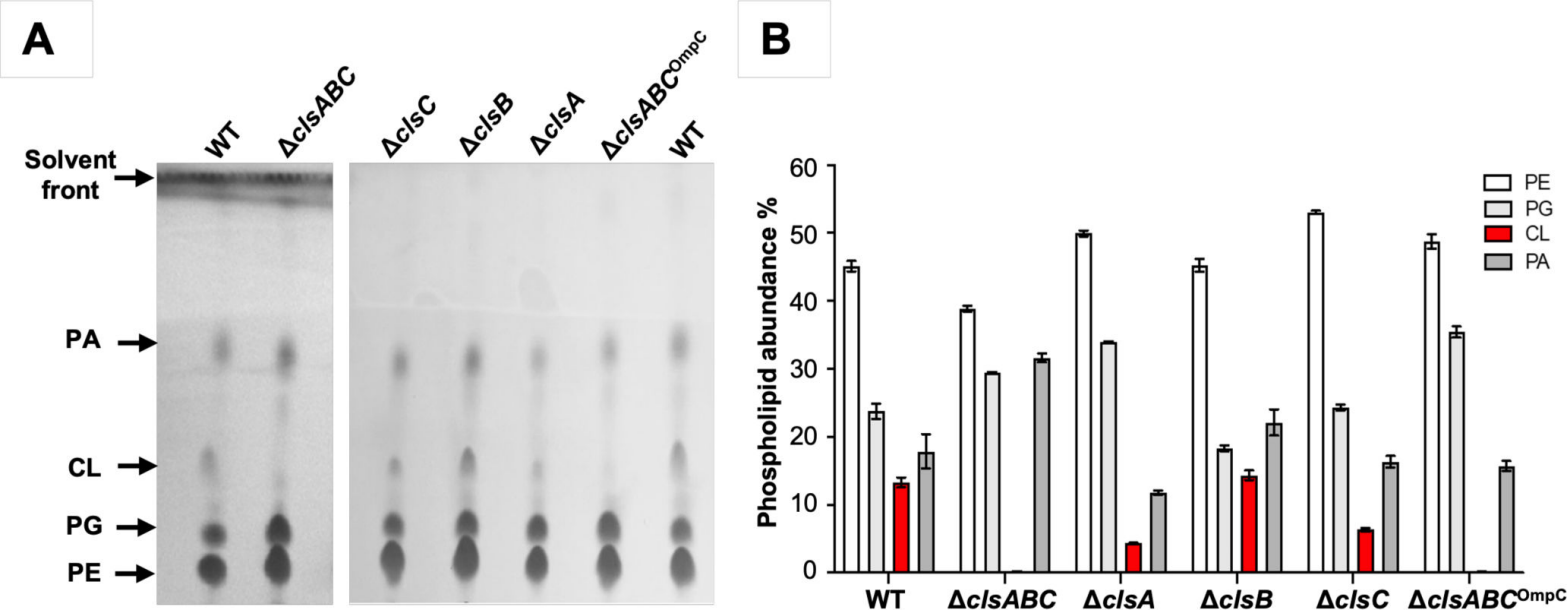
Analysis of lipid content in CL-deficient *E. coli* mutants. (**A**) GPLs were extracted from *E. coli* BW25113, Δ*clsABC,* Δ*clsC,* Δ*clsB,* Δ*clsA,* and Δ*clsABC*^OmpC^. The PLs were separated by TLC using chloroform:hexane:methanol:acetic acid system (50:30:10:5, vol/vol) as the mobile phase. The migration of these species on the silica plate agrees with the migration expected for these PLs in this mobile phase. (**B**) Histograms depict the PL compositions in these strains determined by densitometry assay of PE, PG, CL, and PA against total lipid content from the TLC plates using ImageJ (Fiji) software from three biological replicates.

In order to study differences in gene essentiality between the *E. coli* BW25113 wild-type (WT) and CL synthesis mutants, we first created a suite of isogenic mutants. Therefore, Δ*clsA,* Δ*clsB,* Δ*clsC,* Δ*clsAB,* and Δ*clsABC* were created by successive P1 transduction of the relevant mutated allele from the equivalent Keio library mutant into the parental *E. coli* BW25113 strain. Following each P1-transduction, the kanamycin selection cassette was removed and the deletion confirmed by PCR amplification of the *clsA, clsB,* and *clsC* loci. To confirm the mutants did not synthesize CL, GPLs of these isogenic strains were extracted and analyzed by thin-layer chromatography (TLC) using a chloroform:hexane:methanol:acetic acid solvent system in the ratio of 50:30:10:5 (vol/vol). This solvent system separates GPLs based on polarity and was used because it can clearly differentiate between the four major GPL species present in the *E. coli* cell envelope (2, 19, 20). As expected, there were four visible GPL species present in the WT sample, corresponding to PE, PG, CL, and PA (Fig. 2). However, in the absence of the *clsA* or *clsC* gene, the intensity of CL decreased relative to WT, and CL was absent in *E. coli* BW25113 Δ*clsABC* (Fig. 2), an observation that was consistent with the literature (2).

**Fig 3:**
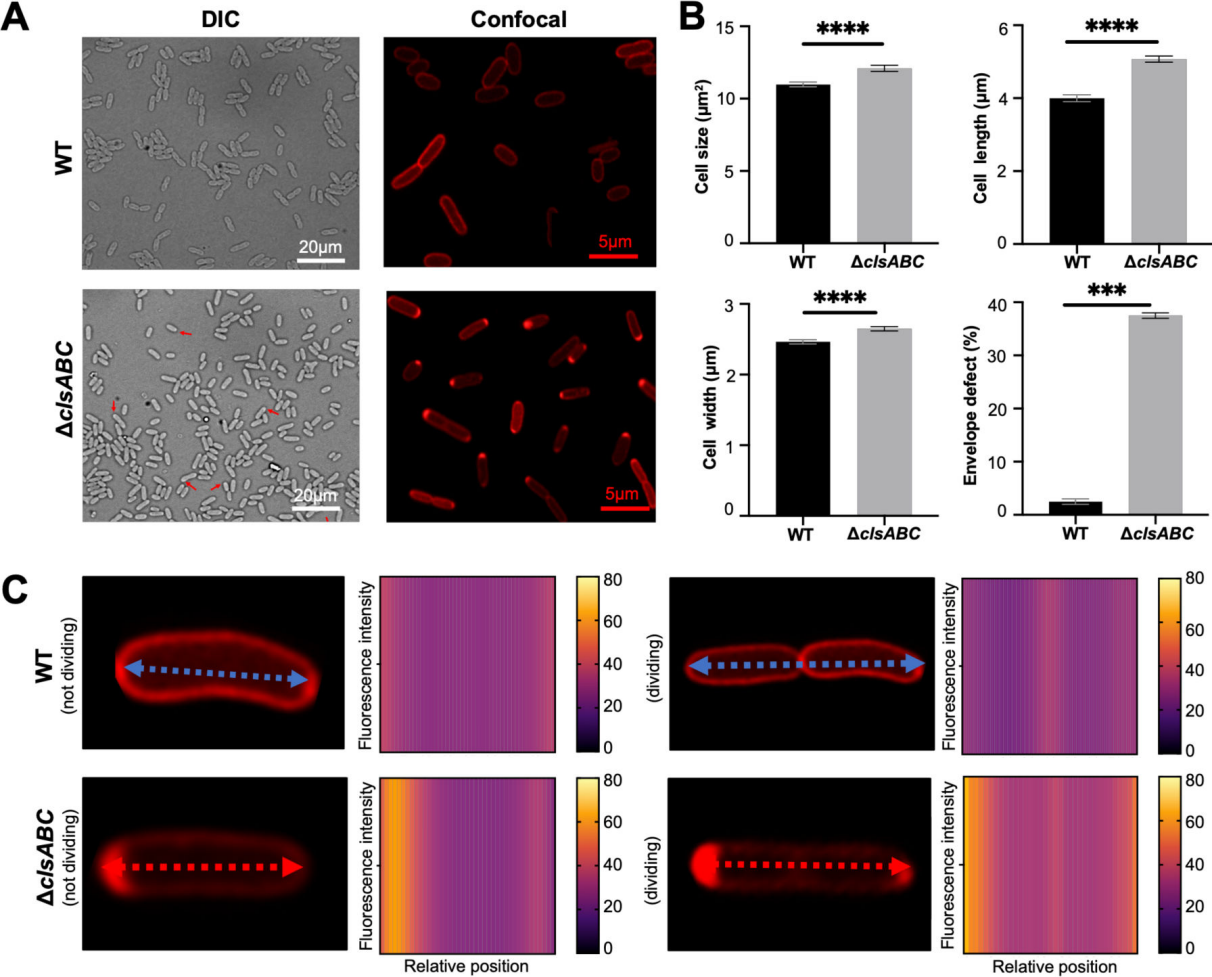
Cell morphology of CL-deficient *E. coli* mutants. (**A**) Representative DIC and confocal microscopy images of *E. coli* BW25113 and *E. coli* BW25113 Δ*clsABC* at stationary phase (after 16 hours growth at 37°C shaking at 180 rpm). The *E. coli* BW25113 Δ*clsABC* harbor the pASK pelB-mcherry. (**B**) Cell area, cell length, cell width, and probability of the envelope defect of *E. coli* BW25113 and *E. coli* BW25113 Δ*clsABC* based on the DIC microscopy images. The average size (area), length, and width of 100 randomly chosen cells were measured using ImageJ (Fiji) software. Cell length was measured along the long axis of the cell, while the cell width was measured perpendicular to the long axis at the widest point of the cell. The cell envelope defect probability represents the percentage of the bacterial cells that had enlarged periplasmic space at cell poles in the 100 chosen cells. Error bars represent standard deviation from the mean. Significance was determined by unpaired Student’s *t*-test (***P < 0.001, ****P < .0001). (**C**) Kymograms of 50 randomly chosen divided or dividing *E. coli* BW25113 and *E. coli* BW25113 Δ*clsABC*. The average fluorescence intensity was measured using ImageJ (Fiji) software and plotted against the position along the long axis of divided or dividing *E. coli* BW25113 and *E. coli* BW25113 Δ*clsABC* cells.

To further probe whether the strains displayed phenotypes associated with CL deficiency, we examined the strains by microscopy. Previous investigations had noted a distinct cellular morphology associated with CL depletion characterized by a “slipper” effect where the IM appears to be detached from the OM at one pole of the cell (2). Therefore, we investigated whether the CL-deficient mutants created here displayed a similar phenotype. The parent strains and the isogenic CL mutants were examined by differential interference contrast (DIC) microscopy during log and stationary phase (Fig. 3A; Fig. S1). We also observed a cell envelope defect and the appearance of an enlarged periplasmic space at cell poles. To further interrogate this phenomenon, a pASK-pelB-mCherry reporter plasmid was transformed into both strains. The fusion of the PelB signal peptide to mCherry resulted in a reporter that allowed visualization of the periplasm by fluorescence microscopy. The red-labeled periplasm of *E. coli* BW25113 and *E. coli* BW25113 Δ*clsABC* harboring pASK-pelB-mCherry plasmid was visualized at both log phase and stationary phase using confocal microscopy (Fig. 3). As expected, mCherry was specifically enriched at one pole of the *clsABC* mutant when compared to the parent strain (Fig. 3). The area, length, and width of 100 randomly selected cells of both strains were measured. The CL mutant was significantly larger for all the metrics, suggesting the mutant cells are longer (5.07 µm vs 4.00 µm) and wider (2.65 µm vs 2.46 µm) than the WT cells (Fig. 3B). Interestingly, measurements of dividing cells indicated that the “detachment” of the IM from the OM occurred at the old pole (Fig. 3C).

Finally, we used whole-genome sequencing to confirm that each CL synthesis gene was deleted appropriately. Interestingly, we noticed that one of our Δ*clsABC* mutants had gained a SNP in *ompC*, which encodes one of the major porins. This SNP (G->A) replaced a glutamine codon (CAG) with a premature stop codon (TAG) at amino acid position 82 (OmpC^Q82X^). Consistent with this finding, Western immunoblotting revealed OmpC was not expressed in this mutant, but it was present in the parent strain, and the individual Δ*clsA*, Δ*clsB*, Δ*clsC* mutants*,* and Δ*clsABC* (Fig. S2). We designated the mutant with the SNP Δ*clsABC*^OmpC^ and subsequently confirmed that this strain lacked CL (Fig. 2) and displayed the similar cellular morphology to the Δ*clsABC* strain described above (Fig. S1). Notably, OmpC expression was not detected in the Δ*clsAB* strain suggesting the SNP was acquired when transducing the *clsB* mutation into the Δ*clsA* mutant during the construction of the triple mutant (Fig. S2).

### Construction of TraDIS libraries

To probe the genetic interactions with the CL synthesis mutants, we created TraDIS libraries in the *E. coli* Δ*clsA,* Δ*clsB,* Δ*clsC,* Δ*clsABC,* and Δ*clsABC*^OmpC^ mutants. Mutants were selected with kanamycin. To identify transposon insertion sites, gDNA of the mutant libraries was extracted for two technical replicates. The transposon-containing fragments were enriched, pooled, and Illumina sequenced (21–23). To accurately identify transposon-gDNA junctions, the Bio-TraDIS toolkit was used to process sequencing reads (24). First, each read was identified using a unique barcode and subsequent specific transposon sequence. After trimming the barcodes and transposon, the filtered reads were aligned to the *E. coli* BW25113 reference genome, and the number of unique insertion sites within each TraDIS library was determined (23). The utility of TraDIS libraries is dependent on the overall density of the transposon library; the more mutants a library has, the more information can be gained during screening, and the more confidence can be derived from the data. Therefore, with the exception of Δ*clsABC*^OmpC^*,* the process was repeated until a library with >500,000 unique insertion points was created for each mutant.

**Fig 4:**
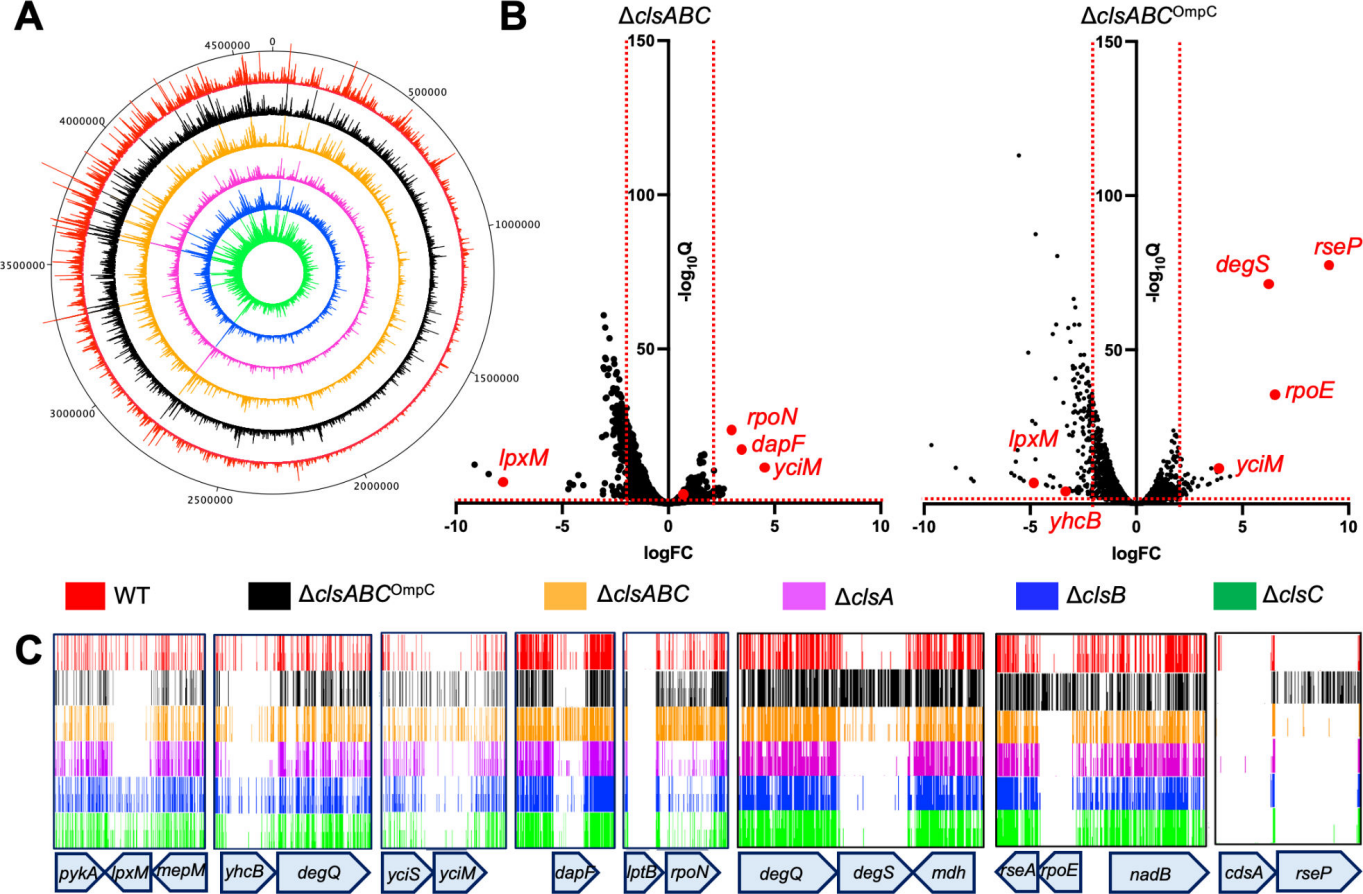
Mapping of transposon insertion sites to *E. coli* BW25113 and isogenic cardiolipin synthesis mutants. (**A**) High-density libraries were generated in the WT *E. coli* BW25113, and isogenic Δ*clsABC* (OmpC^Q82X^), Δ*clsABC*, Δ*clsA*, Δ*clsB*, and Δ*clsC* deletion mutants. Genome-wide visualization of transposon insertion profile in the strains. The inner tracks represent the relative positions of mapped insertions, with the height representing the relative intensity of mapped insertions at that region. (**B**) Volcano plots demonstrating gene fitness scores calculated from the WT TraDIS library compared to the Δ*clsABC*^OmpC^ and Δ*clsABC* libraries. For each point, twofold changes (log_2_FC) of normalized insertion sequencing read abundance per insertion site throughout the BW25113 genome were compared between each TraDIS library, with their respective adjusted *p*-values [*q*-value, represented here as −log_2_(*q*-value)]. (**C**) Transposon insertion profiles for select genes of WT and *cls* deletion mutants. The height of each line in the profile represents the number of transposon insertions (window size: 1–3) at the indicated genome position.

We generated 3,616,158 sequence reads for the *E. coli* BW25113 TraDIS library, revealing 503,333 unique transposon insertion sites (UIPs), giving an average frequency of one insertion every 9.2 bp. Similarly, the libraries generated in the *E. coli* Δ*clsA*, Δ*clsB*, Δ*clsC,* and *clsABC* backgrounds had 646,974, 755,284, 778,082, and 500,060 UIPs, respectively Table S1). In contrast, the *E. coli* Δ*clsABC*^OmpC^ triple mutant had 262,866 UIPs; on discovery of the OmpC SNP, we halted further library construction. However, in all cases, the transposon insertions spanned the entire genome and demonstrated the same *ori-ter* bias previously reported for such libraries (Fig. 4) (21). Notably, for all libraries, there was a good correlation between the number of unique insertions mapped to each gene in the technical replicates, with *R*^2^ values ranging between 0.92 and 0.98 (Fig. S3).

### *rpoE* essentiality in a CL-deficient strain

To identify genes that synthetically interact with the CL synthesis genes, we first performed a bi-modal analysis on our data to identify genes with significantly fewer transposon insertion events (Fig. S4). To minimize biases in the insertion index score, we excluded insertions within the extreme 10% at both the 5′ and 3′ ends of each gene (25). Given that the absence of *clsB* has minimal impact on CL synthesis compared to the parent strain, while the loss of *clsC*, *clsA*, and *clsABC* results in progressively lower CL levels (Fig. 2), we anticipated observing more synthetic interactions with the *clsABC* mutant and fewer, or none, with *clsB*. We expected an intermediate number of interactions for *clsA* and *clsC* mutants. Surprisingly, after comparing the data from the WT, the Δ*clsC*, Δ*clsB*, Δ*clsA,* Δ*clsABC,* and Δ*clsABC*^OmpC^ mutant libraries were predicted to have 35, 66, 71, 59, and 31 essential genes, respectively, which were not essential in the parent (Table S2 and S3). Reassuringly, like Douglass et al. (17), we found *lpxM* was synthetically lethal in the Δ*clsA,* Δ*clsABC,* and Δ*clsABC*^OmpC^ libraries (Fig. 4C). In addition, and as we previously demonstrated (22), *clsA* displayed a synthetically lethal interaction with *yhcB*, which was also evident in the Δ*clsABC* and Δ*clsABC*^OmpC^ mutants (Fig. 4C). However, upon closer inspection, most of the remaining genes had already been identified as essential or their essentiality was designated “unclear” in our prior studies (21).

As the bi-modal analysis method only counts the number of insertions in a given gene, we turned to an alternative analytical method that measures the number of reads for each gene. This method permits genes required for fitness to be distinguished from those required for survival (25). Again, to minimize biases in the insertion index score, we excluded insertions within 10% of the 5′ and 3′ ends of each gene (Table S4). This method also identified *lpxM* and *yhcB* as having synthetic interactions in the Δ*clsA,* Δ*clsABC,* and Δ*clsABC*^OmpC^ libraries (Fig. 4 and Fig. S5). Intriguingly, we observed that three genes, *rpoE*, *degS,* and *rseP,* which belong to the σ^E^ stress response pathway and are essential in the Δ*clsA,* Δ*clsABC,* and parent strains, were not essential in our Δ*clsABC*^OmpC^ library. As this finding was not observed by Douglass *et al.* (17), we hypothesized that the alleviation of the σ^E^ stress response was due to the secondary mutation in *ompC* and that the absence of OmpC and CL bypasses the essentiality of *degS*, *rpoE,* and *rseP* in BW25113 Δ*clsABC*. Interestingly, *yciM (lapB*), *dapF,* and *rpoN* were found to tolerate more insertions in the mutant than the WT suggesting loss of these genes is beneficial to the organism in the absence of CL (Fig. 4).

### Validation of *rpoE* non-essentiality

Our TraDIS data revealed that the normally essential components of the σ^E^ pathway, namely, *rpoE*, *rseP*, and *degS*, were essential in a Δ*clsABC* mutant background, but this essentiality was relieved upon elimination of OmpC expression. To genetically validate these findings, we attempted to create a defined *rpoE* deletion in *E. coli* Δ*clsABC*^OmpC^ and in the isogenic parent strain. As expected, no *rpoE* deletion mutants were recovered from the WT, but Δ*rpoE* mutants were successfully isolated in the Δ*clsABC*^OmpC^ background (Fig. S2). To rule out the possibility that suppressor mutations were responsible for this phenotype, we performed Illumina short-read whole-genome sequencing on genomic DNA from *E. coli* Δ*clsABC*^OmpC^ Δ*rpoE*. After assembly, it was clear that the *E. coli* BW25113 Δ*clsABC*^OmpC^ Δ*rpoE* strain lacked all *cls* genes and *rpoE*, and when compared with the original *E. coli* Δ*clsABC*^OmpC^, no SNPs were detected elsewhere in the genome that could account for the non-essentiality of *rpoE* in this context (Fig. S2). The ability to construct this strain is strong evidence to support the hypothesis that the σ^E^ response is dispensable in the absence of both CL and the major porin OmpC.

**Fig 5:**
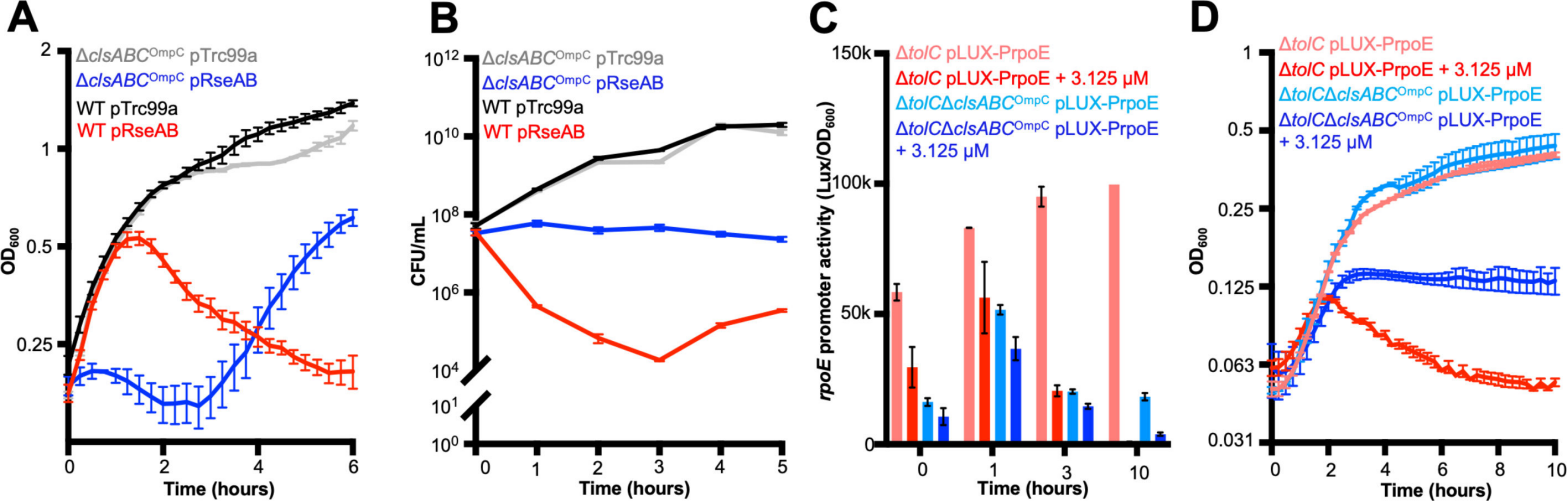
Validation of *rpoE* non-essentiality. (**A**) Growth curves of *E. coli* BW25113 and *E. coli* BW25113 Δ*clsABC*^OmpC^ harboring pRseAB or pTrc99a. Overnight cultures were inoculated into LB supplemented with carbenicillin and grown at 37°C with shaking. The overexpression of *rseAB* was induced with 1 mM IPTG when cultures reached an OD_600_ of 0.1. The OD_600_ growth curve was constructed with OD_600_ readings every 15 minutes and plotted against time in hours. (**B**) CFUs were counted every hour using fresh LB agar supplemented with carbenicillin. (**C**) The *rpoE* promoter activity in *E. coli* BW25113 Δ*tolC* pLUX-P*rpoE* and *E. coli* BW25113 Δ*tolC*Δ*clsABC* OmpC^Q82X^ pLUX-P*rpoE* were measured after incubation for 1, 3, and 10 hours with or without treatment of 3.125 µM batimastat. The *rpoE* promoter activity of the strains was calculated by the following formula: luminescence intensity/OD_600_. (**D**) The OD_600_ growth curves of both strains were constructed with OD_600_ readings every 15 minutes and plotted against time in hours with or without treatment of 3.125 µM batimastat. Error bars represent the standard deviation between three biological replicates.

As we were unable to generate an *rpoE* mutation in either *E. coli* BW25113 or the Δ*clsABC*, we employed an alternative strategy to test our model. Previous studies have demonstrated that overexpression of RseA and RseB is lethal due to their sequestration of σ^E^ at the IM thereby preventing σ^E^-dependent transcription (26). However, based on our observations above, we hypothesized that overexpression of RseA and RseB in *E. coli* Δ*clsABC*^OmpC^ would be tolerated, whereas it would exhibit a lethal effect in the isogenic parent strain. To test the hypothesis, both strains were transformed with the pRseAB plasmid, which encodes RseA and RseB under the control of an IPTG-inducible *trc* promoter, or with the corresponding empty vector, pTrc99a (26). Overnight cultures of these strains were transferred to fresh LB broth supplemented with carbenicillin and grown at 37°C. Overexpression of *rseA* and *rseB* was induced at an OD_600_ of 0.1 by the addition of 1 mM IPTG and the optical density of the cultures was monitored over time. As previously observed, overexpression of RseA and RseB, and the consequent sequestration of σ^E^, resulted in a decrease in the optical density of the WT strain after 2.5 generations (Fig. 5). In contrast, Δ*clsABC*^OmpC^ pRseAB exhibited a longer lag phase (~3 hours) but kept growing. The growth of the WT and triple mutant empty vector controls was comparable (Fig. 5).

However, given morphological variations can affect optical density tests, and that the Δ*clsABC* mutants display morphological variation, colony-forming units (CFU) counts on LB agar plates supplemented with carbenicillin for vector maintenance were used to calculate the number of viable cells. As expected, the presence of the pTrc99a vector alone did not have any effect on the growth of either the parent strain or the Δ*clsABC*^OmpC^ mutant. However, 2 hours after IPTG addition, the CFUs of *E. coli* BW25113 pRseAB decreased by two orders of magnitude (Fig. 5). Surprisingly, while overexpression of RseA and RseB did not adversely affect cell viability in the Δ*clsABC*^OmpC^ pRseAB strain, in contrast to the controls and to the WT, the number of CFUs of this strain remained stable throughout the experiment. These observations suggest that sequestration of RpoE leads to more pronounced growth defects in *E. coli* BW25113 WT compared to the Δ*clsABC*^OmpC^ triple mutant and supports the hypothesis that *rpoE* is not essential in a strain lacking CL and major porins.

To further validate the non-essentiality of *rpoE*, we employed a chemical inactivation approach as an orthogonal strategy. Batimastat, a compound known to inhibit the activity of RseP, prevents the degradation of RseA, effectively sequestering σ^E^ at the IM and preventing activation of the σ^E^ regulon (27). We hypothesized that batimastat would inhibit the growth of the parent strain, but not that of the Δ*clsABC*^OmpC^ mutant. However, previous reports have indicated that batimastat is actively effluxed from cells via TolC (27) and, therefore, to mitigate this efflux, we constructed the *E. coli* BW25113 Δ*tolC* and BW25113 Δ*tolC*Δ*clsABC*^OmpC^ strains using the Datsenko and Wanner method. To evaluate the impact of batimastat on the activation of the σ^E^ regulon, we measured the activity of the *rpoE* promoter in these strains. The *rpoE* promoter was cloned into the pLUX plasmid, generating pLUX-PrpoE, where the luciferase operon was placed under the control of σ^E^. Subsequently, *E. coli* Δ*tolC* and Δ*tolC*Δ*clsABC*^OmpC^ were transformed with either pLUX-P*rpoE* or pLux. For each strain, the total luminescence intensity was monitored over a 10 hour period at 37°C, and OD_600_ was enumerated. The ratio of luminescence to OD_600_ (Lux/OD) was used to calculate the *rpoE* promoter activity. The activity of the *rpoE* promoter was significantly greater in the *E. coli* Δ*tolC* compared with the Δ*tolC*Δ*clsABC*^OmpC^ mutant (Fig. 5), suggesting the basal level of σ^E^ activity was elevated in the former strain. This finding implies that the concurrent loss of CL synthesis and major porin production in the mutant suppressed σ^E^ activation. Importantly, the OD_600_ of *E. coli* BW25113 Δ*tolC* peaked 2 hours after the addition of batimastat and subsequently decreased over the following 10 hours, indicating that the inhibition of σ^E^ activation was lethal in this strain. In contrast, batimastat did not induce a lethal effect in *E. coli* BW25113 Δ*tolC*Δ*clsABC*^OmpC^ (Fig. 5). In conclusion, the inhibition of σ^E^ activity resulted in growth defects in *E. coli* Δ*tolC*, but to a far lesser extent in *E. coli* Δ*tolC*Δ*clsABC*. These data further support the hypothesis that *rpoE* is not essential in a mutant lacking CL and the major porins.

### Sec activity and *rpoE* non-essentiality

**Fig 6:**
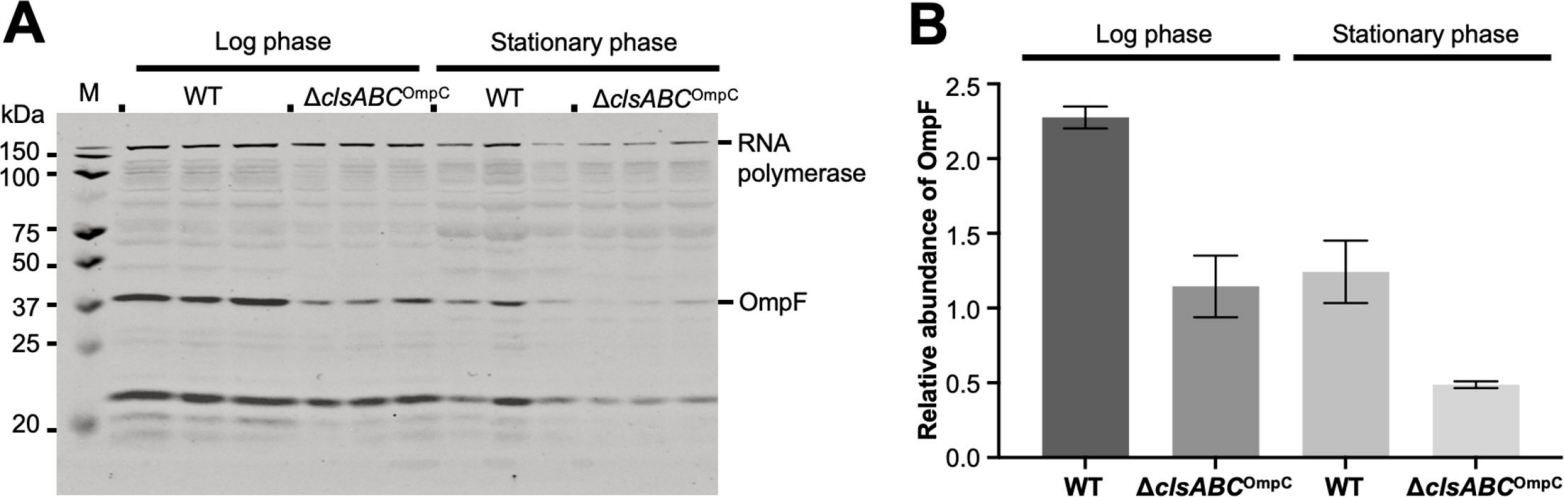
Detection of OmpF in CL-deficient mutants. (**A**) Whole cell lysates extracted from log and stationary phase bacteria separated by 12% SDS-PAGE. The gel was immunoblotted with anti-RNA polymerase and anti-OmpF primary antibodies. The molecular weight of OmpF is approximately 37 kDa. (**B**) Histograms depict the abundance of OmpF in the strains determined by densitometry assay OmpF against RNA polymerase (loading control) using ImageJ (Fiji) software from three biological replicates.

Previous data demonstrated that the simultaneous deletion of two genes encoding major OMPs, OmpA and OmpC, could relieve *rpoE* essentiality in *E. coli,* but that deletion of either gene independently did not (9). As the σ^E^ stress response is essential for bacterial viability, because it can sense and prevent the toxic accumulation of misfolded or unfolded OMPs in the periplasm (6, 28), we hypothesized that like the *E. coli* Δ*ompA*Δ*ompC* mutant, the CL mutant might have decreased levels of OMPs. To test this hypothesis, the abundance of two major OMPs (OmpC and OmpF) in the parental strain and the isogenic CL mutant was measured using immunoblot analysis. The strains were grown in LB broth at 37°C with shaking (180 rpm). Whole cell lysate samples were taken at different growth phases (log phase and stationary phase). Protein samples were separated by SDS-PAGE, prior to being stained with Coomassie or transferred to nitrocellulose for immunoblotting using OmpC antibody or OmpF antibody. We found OmpC was absent in *E. coli* BW25113 Δ*clsABC*^OmpC^ as expected (Fig S2). However, the level of OmpF in the CL mutant was lower compared to parent strain in both log and stationary phase (Fig. 6). Based on these results, we hypothesized that CL deficiency decreases the total levels of OmpC and OmpF in the extracytoplasmic compartment.

**Fig 7:**
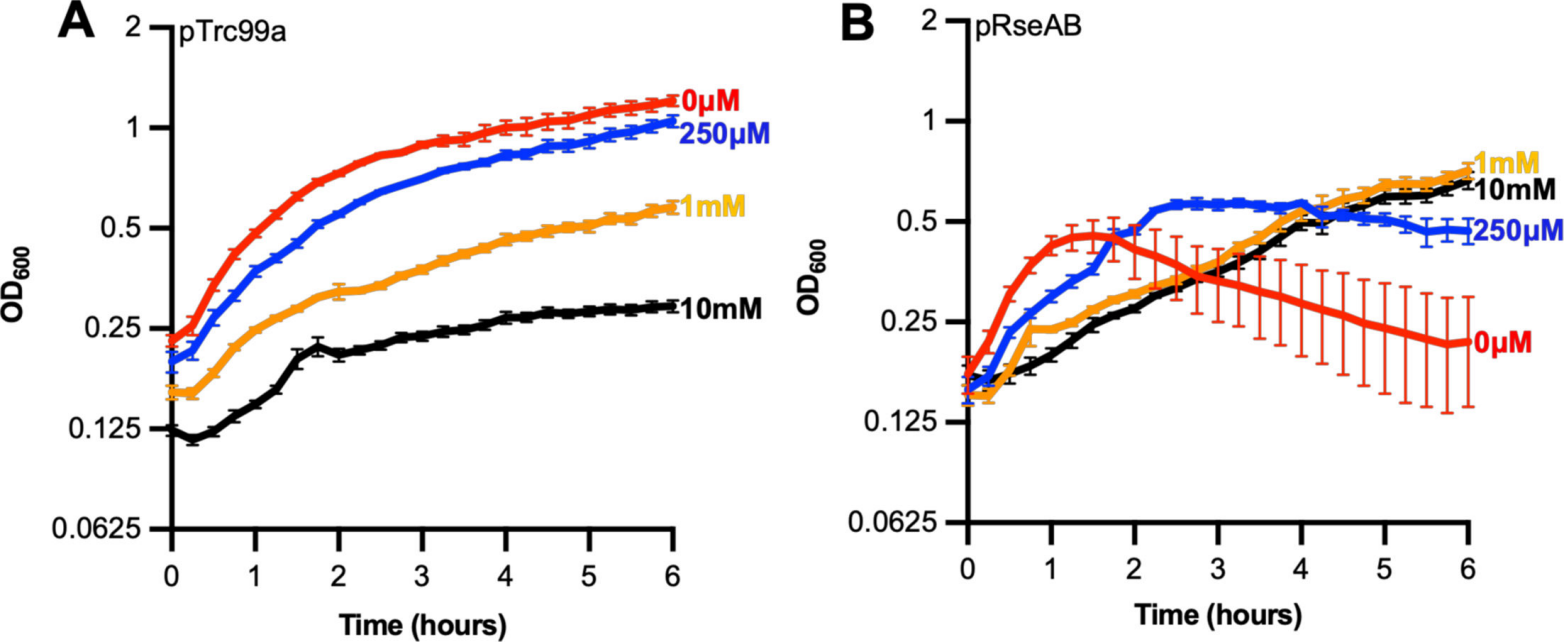
Sodium azide treatment of *E. coli* BW25113 pRseAB and *E. coli* BW25113 pTrc99a. Triplicate overnight cultures were inoculated into LB supplemented with carbenicillin and grown at 37°C with aeration. Expression of *rseAB* was induced with 1 mM IPTG when cultures reached an OD_600_ of 0.1 and treated with NaN_3_ to varying concentrations of 0 µM, 250 µM, 1 mM, and 10 mM. The growth curves of biological triplicates of *E. coli* BW25113 pTrc99a (**A**) and pRseAB (**B**) were plotted with the mean OD_600_ readings every 15 minutes and plotted against time in hours. Error bars represent the standard deviation between three biological replicates.

OMPs are synthesized in the cytoplasm and translocated across the IM via the Sec translocon (29). Previously, it was reported that the function of the Sec translocon depends on the presence of CL (12, 30) and that the biogenesis of OmpF via the Sec pathway was impaired in CL-depleted *E. coli* (30). Therefore, we hypothesized that the decreased abundance of OMPs in the CL deficient mutants is due to decreased Sec activity. In such a situation, *rpoE* would become non-essential if the activity of the Sec pathway was reduced, concomitantly decreasing the level of misfolded and unfolded OMPs in the periplasm. To determine if this is the case, *E. coli* BW25113 was treated with sodium azide (NaN_3_; a SecA ATPase inhibitor) to inhibit Sec activity (31). Cultures of *E. coli* BW25113 pRseAB and *E. coli* BW25113 pTrc99a (the empty vector) were grown to an OD_600_ of 0.1, RseAB overexpression was induced with 1 mM IPTG with different concentrations of NaN_3_. Subsequently, the cultures were grown at 37°C with shaking (180 rpm) for 10 hours, and the optical density of the cultures was monitored (Fig. 7). The decrease in optical density of *E. coli* BW25113 pRseAB between 1.5 and 6 hours was inversely related to the increase of NaN_3_ concentration. Additionally, the growth of *E. coli* BW25113 pRseAB and *E. coli* BW25113 pTrc99a was comparable in the presence of 1 mM NaN_3_ (Fig. 7). Notably, with the addition of 10 mM NaN_3_, *E. coli* BW25113 pRseAB grew at a greater rate than the empty vector control. It was concluded that reduced Sec activity (through sodium azide treatment) alleviates the lethality of *rpoE* sequestration through RseAB overexpression.

## DISCUSSION

Despite extensive research on the synthesis and biochemical properties of *E. coli* GPLs, the specific biological functions of each major GPL remain poorly understood. Although CL constitutes approximately 5% of the GPLs in *E. coli*, it is not essential for growth under standard laboratory conditions. Nonetheless, the significance of CL as a membrane lipid is underscored by several known factors: (i) *E. coli* employs three distinct synthases for its production; (ii) CL synthesis is conserved across the bacterial phylogeny, whereas the synthesis of PE, which accounts for the majority (~70%) of GPLs in *E. coli*, is not; and (iii) the retention of CL synthesis in mitochondria, which evolved from an endosymbiotic alpha-proteobacterium. Indeed, it is widely accepted that CL plays a crucial role in maintaining the structural integrity and morphology of the mitochondrial membranes, as well as regulating the activity of various proteins and enzymes in bacteria such as the polar localization of ProP and protein transport through the Sec apparatus (32). Here, we aimed to identify genes that become essential in the absence of CL. Our findings confirm the synthetic lethal interactions between CL synthesis and *lpxM,* and CL synthesis and *yhcB,* observed previously (22). Our data suggest that depletion of *lapB* which regulates LPS synthesis improved the fitness of a CL-deficient mutant and also demonstrated a connection between CL production, Sec-dependent protein secretion, and the essential σ^E^ response.

LpxM is an acyltransferase that plays a critical role in the final stage of lipid A biosynthesis, by acylating Kdo2-lipid Iva to create hexa-acylated LPS (18). Several studies have demonstrated that underacylated lipid A species, such as those arising from loss of the acyltransferases LpxM or LpxL, are poorly translocated by the MsbA flippase. Based on observations from several groups, it was hypothesized that CL promotes the transport of lipid A substrates by MsbA by aiding in MsbA ATPase activity, and hence the concomitant loss of CL and LpxM leads to a lethal accumulation of LPS in the IM (33, 34) . Another protein involved in LPS biogenesis, LapB, is essential for viability under laboratory growth where it recruits FtsH to mediate proteolysis of LpxC, the enzyme responsible for the first committed step in LPS biogenesis. Therefore, loss of LapB is hypothesized to result in a lethal accumulation of LPS in the IM (35). Given this hypothesis, it is then surprising to see increased insertion frequencies in *lapB* (*yciM*), as one might expect this to phenocopy the synthetic lethal relationship between *clsA* and *lpxM*. However, recent data have revealed that LapB also serves as a protein-protein interaction hub for key enzymes in GPL biogenesis, and multicopy suppressors of *lapB* essentiality include *fabZ* and *fabB* which are involved in fatty acid biogenesis (36, 37).

Previously, we demonstrated that the loss of either *clsA* or *lpxM* was deleterious in a *yhcB* mutant background, suggesting a synthetic interaction between these loci (22). In the present study, we show that deletion of *clsA* and all three *clsABC* genes also have a synthetic interaction with *yhcB*, thereby corroborating our earlier findings. YhcB has been implicated in the coordination of GPL, LPS, and peptidoglycan biogenesis, with *yhcB* deletion mutants exhibiting pronounced morphological abnormalities, including defects in cell shape and size. Additionally, work by others revealed that YhcB also acted as a protein:protein interaction hub, with physical interactions occurring with the elongasome and the LapB partner protein, LapA (38). In our previous analysis, we found that mutations impairing fatty acid biosynthesis, as well as mutations that result in upregulation of *relA,* encoding the (p)ppGpp synthase, were capable of suppressing the Δ*yhcB* phenotype (22). The alarmone (p)ppGpp is a key regulator of cell physiology, known to modulate cell size and shape by downregulating phospholipid and protein biosynthesis pathways (39). Similar observations were subsequently made by Stanley and Trent (40); however, the precise reason for the synthetic interactions is not known. One possible explanation is that the absence of CL in a Δ*yhcB* background permits increased fatty acid flux toward membrane phospholipid synthesis. Supporting this hypothesis, it has been reported that CL deficiency results in elevated (p)ppGpp levels, indicating that the cell may be trying to compensate for excess GPL or free fatty acids (2). In this context, it is notable that the Δ*clsABC* mutant appeared to contain higher levels of phosphatidic acid, the universal precursor to GPL synthesis. However, due to the extensive metabolic interdependence of fatty acid and GPL biosynthetic pathways, disentangling the individual contributions of each pathway to the observed phenotypes is challenging. Modulation of one pathway invariably perturbs the other, making it experimentally difficult to isolate their effects. An alternative explanation is that protein and/or LPS production is disrupted to an extent that the OM to IM ratio cannot be maintained at a level consistent with viability. However, it may also simply be that loss of CL dampens the σ^E^ response, which is vital for survival of Δ*yhcB* mutants due to their dysregulated cell envelope biosynthesis

In agreement with the hypothesis that loss of CL results in disruption to coordinated GPL, LPS, and protein biosynthesis, our data identified that the σ^E^ stress response was not essential in a background lacking in the major porin OmpC and CL. The observation was validated by genetic and phenotypic assays, demonstrating CL mutants produced less OmpF than the parent strain, had decreased *rpoE* expression, and upon σ^E^ sequestration the growth defect was significantly reduced in a CL mutant when compared to parent strain. Previously, experiments demonstrated that the transcription of σ^E^-controlled genes were not significantly activated in the absence of CL (41). The essentiality of the σ^E^ stress response is partially dependent on the presence of major OMPs. It has previously been reported that an RseP depletion strain can survive in the absence of both OmpA and OmpC (9). As the previous study reported that a Δ*rseP* allele could only be introduced into a double Δ*ompA*Δ*ompC* mutant, but not into Δ*ompC* or Δ*ompA* single mutants, this suggests that the premature stop codon in *ompC* alone is insufficient for bypassing *rpoE* essentiality (9). Interestingly, attempts to construct the Δ*clsABC* were repeatedly confounded by the appearance of SNPs in genes encoding OMPs (data not shown), including the *yehABCD* operon, which encodes a fimbrial structure produced in most of the *E. coli* strains (42). This suggests that the reduction of OMP production may be beneficial in a CL minus background. Together these data suggest a new paradigm for the bypass of *rpoE* essentiality, through the combined absence of major OMPs and CL.

**Fig 8:**
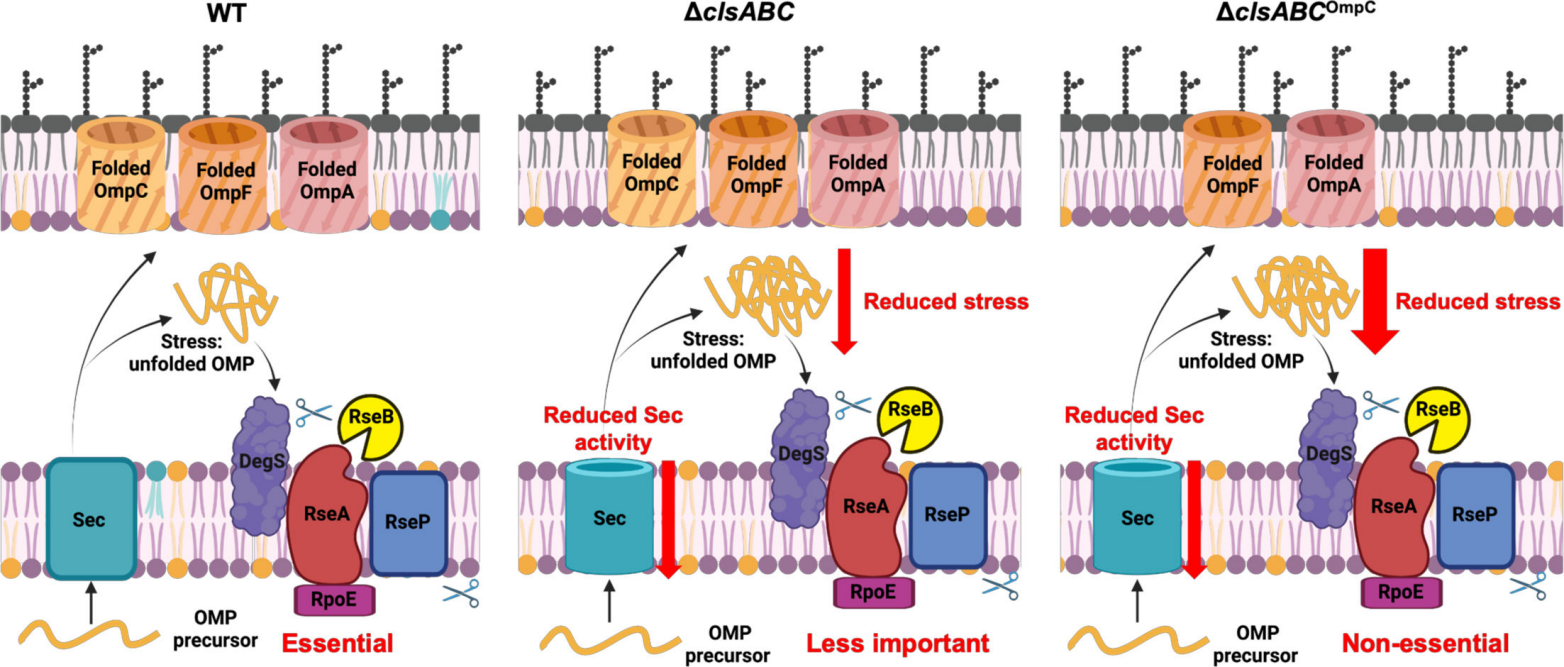
Schematic diagrams of *rpoE* essentiality in *E. coli* BW25113, Δ*clsABC,* and Δ*clsABC*^OmpC^. The phospholipids labeled in purple, yellow, and blue represent PE, PG, and CL, respectively. The red downward arrows represent the decreased Sec activity, decreased density uOMPs in periplasm, or decreased level of folded OMP in OM.

Nevertheless, consistent with our hypothesis that reduced OMP biogenesis is beneficial in a CL negative background, our studies suggested that CL deficiency decreased OMP production. Importantly, the function of the Sec translocon depends on the presence of CL (12). CL associates with the SecYEG protein channel complex in *E. coli* binding to the K115 and K181 residues of SecY, and stabilizing SecYEG dimerization (12, 43). Additionally, CL creates a high-affinity binding surface for the 25 N-terminal residues of SecA ATPase (12, 43). SecA protein serves as a motor protein that not only directs the pre-secretory proteins to the membrane integrated protein conducting channel SecYEG but also provides the energy for protein translocation via ATP hydrolysis (44). In the absence of CL, the SecYEG should be less stable, and consequently, protein secretion across the IM to the periplasm will be reduced. Our model suggests that such a reduction of protein translocation to the periplasm reduces the accumulation of unfolded proteins in the periplasm, relieving the need for the σ^E^ extracytoplasmic stress response. In support of this, *rpoE* essentiality could be relieved by treating cells with the SecA inhibitor sodium azide (Fig. 8).

There are some cautionary lessons to be learned from this study. While TraDIS and related TIS techniques offer a powerful method for genome-wide analysis of gene function, several limitations are highlighted here. First, the statistical robustness of any transposon insertion sequencing approach is inherently dependent on the density of the library. As could be observed in this study, libraries with fewer unique insertion points are less likely to yield statistically reliable data, resulting in an increased incidence of false positives. Second, even a single SNP can significantly skew the outcomes of transposon insertion sequencing experiments. Although this may occasionally lead to biologically relevant insights, as demonstrated in this study, it also introduces the potential for misleading conclusions if the parental strain is not confirmed by whole genome sequencing. Nevertheless, despite these limitations, TraDIS and its associated TIS techniques remain formidable and invaluable tools for investigating biological complexity on a genomic scale.

In summary, the loss of CL disrupts multiple pathways involved in cell envelope biogenesis; however, this perturbation does not activate the σ^E^-mediated envelope stress response. The underlying mechanism by which CL deficiency alters cell morphology, particularly the selective enlargement of the periplasmic space at the old pole, remains unclear. Moreover, the observation that the σ^E^ stress response is not universally essential among bacterial species, including the closely related *S. enterica*, suggests species-specific variations in stress response pathways that are not yet fully understood (45). This study lays the groundwork for future investigations into the molecular basis of σ^E^ essentiality and its evolutionary divergence across species. Ultimately, although further research is required to elucidate the precise molecular consequences of CL depletion, the findings underscore the critical role of coordinated synthesis of proteins, glycerophospholipids (GPLs), and lipopolysaccharides (LPS) in preserving cell envelope integrity.

## MATERIALS AND METHODS

### Bacterial strains and growth conditions

Bacterial strains used in this study are listed in Table S5. Strains were routinely cultivated on solid lysogeny broth (LB) agar (10 g/L tryptone, 5 g/L yeast extract, 10 g/L NaCl, 15 g/L agar) or in liquid LB (10 g/L tryptone, 5 g/L yeast extract, 10 g/L NaCl). For transposon library construction, electro-competent cells were grown in 2 × YT broth (5 g/L NaCl, 16 g/L tryptone, and 10 g/L yeast extract). Super Optimal broth with Catabolite repression (SOC) medium (Sigma Aldrich) was used in the recovery step during electroporation transformations to increase transformation efficiency. Unless otherwise stated, strains were aerobically grown in LB at 37°C under shaking conditions (180 rpm). Culture media were supplemented with antibiotics or other supplements as required. The final antibiotic concentrations for culturing bacteria were as follows: 100  µg/mL kanamycin, 100 µg/mL carbenicillin, or 35 µg/mL chloramphenicol. When required, media were supplemented with 0.4% (wt/vol) L-arabinose or 0.4% (wt/vol) glucose for expression or repression of genes regulated by the arabinose inducible promoter. For OD_600_ growth curve determination, 200 µL of the cultures was then added into each well of a 96-well microtiter plate (U-bottom, Greiner Bio-One), covered by a sterile, Gas Permeable Sealing Membrane (Diversified Biotech), and then incubated statically in a POLARstar Omega Multimode Microplate Reader (BMG LABTECH) for 17 h at 37°C, where OD_600_ readings were taken every 15 minutes. Bacterial growth curve was plotted using GraphPad Prism V10.

### Molecular biology

Genomic DNA (gDNA) was extracted from overnight cultures using the RTP Bacteria DNA Mini Kit (STRATEC Molecular) as per manufacturer’s instruction. The isolated genomic material was stored at 4°C. Plasmids used in this study are detailed in Table S6. Plasmids were prepared from overnight cultures using the Qiagen mini prep kit (Qiagen) according to the manufacturer’s instructions. DNA was amplified using Q5 High-Fidelity 2X Master Mix (New England Biolabs) or MyTaq Red Mix (Bioline) according to the manufacturer’s instructions using the primers listed in Table S7. Amplified products were purified using the Qiagen PCR purification kit (Qiagen) as per manufacturer’s instructions. DNA was quantified using Nanodrop One (Thermo Fisher Scientific) following the manufacturer’s protocol. For the gDNA extracted for Illumina sequencing, samples quantified using the QubitTM dsDNA HS Assay kit (Invitrogen) as per manufacturer’s instructions. DNA samples were diluted with 6× DNA Loading Dye (Life Technologies) and loaded onto 1% agarose-TAE gels containing 1/30,000 vol of SYBR Safe DNA gel stain (Life Technologies). DNA fragments were separated in 1× TAE buffer (1 mM Disodium EDTA salt dihydrate, 40 mM Tris-base, and 20 mM Acetic Acid) at 100 V for 30 min prior to being visualized under UV transilluminator (BIO-RAD).

For gene cloning experiments, DNA inserts were ligated into the digested plasmid (50 ng) using T4 DNA Ligase (New England BioLabs) and transformed into 50 µL of NEB 5-alpha Competent *E. coli* (high efficiency) cells before selection on LB agar with required antibiotics, as previously described. For gene deletions, target genes were replaced with a linear DNA fragment containing a kanamycin resistance cassette (aminoglycoside phosphotransferase encoded by *aph*) by λ Red recombination according to the protocol described by Datsenko and Wanner (46) or by P1 transduction of mutated alleles from KEIO library mutants, as previously described (47). Mutants were selected on LB agar plates supplemented with the required antibiotic and successful mutant construction was confirmed by PCR. The pASK-pelB-mcherry plasmid was generated from components of previously described plasmids (48, 49).

### TraDIS library construction and data analyses

TraDIS libraries were constructed essentially as we have described previously (21–23). Briefly, the EZ-Tn5 Transposon was introduced into *E. coli* K-12 strain BW25113 by electrotransformation. Successful transformants were selected on medium supplemented with kanamycin. After selection, surviving bacterial cells were pooled and resuspended in 30% glycerol in LB for storage at −80°C. Subsequently, genomic DNA was extracted, fragmented by ultrasonication to an average size of ~250 bp, and prepared for sequencing using the NEB Next Ultra I kit (E7370L, NEB). Multiple rounds of PCR were performed to amplify the transposon-genome junction region and to add nucleotide barcodes sequences and adapters for sequencing. The final product was purified using SPRI beads and then quantified by qPCR following the kit instructions (KAPA Library Quantification Kit, Roche). Fragments were sequenced using an Illumina MiSeq using v3 150 cycle cartridges (MS-102-3001, Illumina), with PhiX loading control (Illumina).

Sequencing data were processed using a previously described approach (23). In brief, sequencing reads were first filtered by the Illumina barcode on BaseSpace (Illumina). Subsequently, the inline-index barcode for each replicate was trimmed using the FastX_toolkit. The Trimmomatic-0.36-6 tool was then used to remove reads shorter than 20 bp (50). The remaining reads were aligned to the *E. coli BW25113* reference genome (CP009273.1) using the bwa mem algorithm (51). The position and frequency of transposon insertions in the library were determined using customized python scripts. The position_depth_count output file that represents the diversity of transposon mutants was manually inspected via Artemis genome browser (52). Using the position_depth_count output file as an input, the insertion index score for each gene was calculated using the tradis_gene_insert_sites script from Bio-TraDIS toolkit. Linear correlation of the insertion index scores of individual genes between sequencing technical replicates was calculated using Graphpad Prism V10. The essential genes in each TraDIS library were predicted by Bi-modal analysis using the tradis_essentiality.R script from Bio-TraDIS toolkit. Log fold changes of the gene sequencing coverages between TraDIS libraries were then measured by comparative analysis using the tradis_comparison.R script from the Bio-TraDIS toolkit and plotted on a volcano plot using Graphpad Prism V10.

### Protein analyses

Cellular fractions were prepared and analyzed by Tris-glycine SDS-PAGE and Western immunoblotting as we have described previously (53). Briefly, 1 mm thick 10% (vol/vol) acrylamide gels were made using a Mini-PROTEAN Tetra Handcast System, and samples were separated by applying a 50 V for 15 min followed by 120 V for 2 h. Coomassie Brilliant Blue staining was used to visualize SDS-PAGE gels, and gels were imaged on a Bio-Rad ChemiDoc MP. For western blotting, SDS-PAGE gels were transferred to nitrocellulose membranes using the Trans-Blot Turbo System (Bio-Rad). The program settings used for the transfer were 1.3 A, 25 V for 7 min. After incubation for 30 min at RT with shaking in blocking buffer, the primary was added and incubation continued for 1 hour. Subsequently, the blots were washed, and secondary antibodies were added and incubation continued for 1 h. Antibodies used in this study are listed in Table S8. After washing, fluorescence signals from the secondary antibodies were detected using Odyssey CLx imaging system. In both cases, the PageRuler protein ladder was used to estimate protein size.

### Phospholipid analyses

To extract the phospholipids of bacterial membranes, a modified version of the “Bligh and Dyer” protocol was followed (54). Glass implements were used for the entirety of the protocol to avoid plastic contamination of the chloroform. Volume of 20 mL bacterial overnight culture was centrifuged at 2,470 × *g*, 4°C for 10 min. The harvested pellets were resuspended in 1 mL of dH_2_O and transferred to glass test tubes. Following this, 1.25 mL chloroform and 2.5 mL methanol were added to each tube and vortexed to produce a single phase. The tubes were incubated at 50°C for 30 min prior to further addition of 1.25 mL chloroform and 1.25 mL dH_2_O. The tubes were vortexed and centrifuged at 200 × *g* for 10 min to generate a two-phase solution. After centrifugation, the bottom phase in the tubes containing the lipid samples was extracted using glass Pasteur pipettes and transferred to fresh test tubes. For authentic upper phase preparation, 2.25 mL dH_2_O, 2.5 mL chloroform, and 2.5 mL methanol were added into each fresh glass test tube. The tubes were vortexed and centrifuged at 200 × *g* for 10 min to generate a two-phase solution. The upper phase (authentic upper phase) was then extracted using glass Pasteur pipettes and transferred to fresh glass collection tubes. Following this, 2.5 mL of the authentic upper phase was added into each extracted lipid sample. The samples were vortexed before being centrifuged at 200 × *g* for 10 min. The bottom phase was extracted and transferred to a fresh test tube prior to drying the sample under a stream of nitrogen gas at 40°C. The final extracted lipids were resuspended in 200 µL chloroform and stored at −30°C.

Thin-layer chromatography (TLC) was done as previously described (22). Briefly, 10 µL of the phospholipid chloroform solution was loaded on a TLC silica gel 60 F_254_ (Merck) by capillary tubes. The TLC plate was dried at 56°C for 15 minutes before being placed in a solvent tank with chloroform:hexane:methanol:acetic acid (50:30:10:5, vol/vol) (2). After the mobile phase reached 1 cm from the top of the plate, the plate was removed from the solvent system and left to dry. The dried plate was dipped into 10% (wt/vol) phosphomolybdic acid (PMA) in ethanol for phospholipid staining and further visualized by charring the plate with a heat gun.

### Luciferase assay

To measure the *rpoE* promoter activity in *E. coli*, a luciferase reporter plasmid, pLUX-*rpoE,* was used. This plasmid contained the *rpoE* promoter region upstream of the bioluminescence *luxCDABE* genes. pLUX-*rpoE* and pLUX (empty vector control) were first transformed into pre-prepared competent cells. Overnight cultures of the strains, harboring pLUX-*rpoE* or an pLUX, were diluted into fresh LB broth supplemented with kanamycin to an OD_600_ of 0.05. For each diluted culture, 100 µL was transferred to a well of a Black/Clear bottom tissue culture treated 96-well plate (Corning Incorporated). The OD_600_ and total luminescence intensity were monitored in parallel at 37°C for 3 h using a microplate reader (TECAN). The amount of luciferase produced under the control of the *rpoE* promoter of each culture was calculated by the following formula: luminescence intensity/OD_600_.

### Microscopy

Bacterial strains were grown in LB broth. Samples of both exponential phase (OD_600_ of 0.4) and stationary phase (after overnight growth) were collected. Cultures were centrifuged at 3,200 × *g* for 2 min and resuspended in 1 mL PBS. Before visualization, membranes were stained using the membrane dye FM 4-64FX (5 µg/mL), while the nucleic acid was stained using DAPI (4′,6-diamidino-2-phenylindole, 300 nM). A coverslip sized 0.5% (wt/vol) agarose pad was made with TAE buffer and placed onto the middle of a glass slide. A volume of 5 µL resuspended culture was added onto the agarose pad and covered with a coverslip. Nail polish was then used to seal the gap between the coverslip and the glass slide around the agarose pad before imaging. Widefield images were acquired by Differential Interference Contrast (DIC) microscopy using an Upright Widefield Microscopes Fluroro 2 (Axiolmager) (60×/1.40 OIL) with Zeiss Zen Blue 3 with Tiling and EDF modules software. Confocal microscopy was done using an Inverted LSM 880 Fast Airyscan (63×/1.40 OIL) with Zeiss Zen 2012 Black software. For images analysis, ImageJ (Fiji) was used to measure the cell size and length. For data visualization, Graphpad Prism V10 was used for plotting.

## SUPPLEMENTAL MATERIAL

Supplemental Figures mbio.01613-25-s0001.docx Figures S1 to S5.

Tables S1 and S2 mbio.01613-25-s0002.docx Summary of and uniquely essential genes in TraDIS libraries.

Tables S5 to S8 mbio.01613-25-s0003.docx Strains, plasmids, primers, and antibodies.

Table S3 mbio.01613-25-s0004.xlsx Bimodal analysis of WT gene essentiality.

Table S4 mbio.01613-25-s0005.xlsx Gene fitness scores.

ASM does not own the copyrights to Supplemental Material that may be linked to, or accessed through, an article. The authors have granted ASM a non-exclusive, world-wide license to publish the Supplemental Material files. Please contact the corresponding author directly for reuse.
