# Supplementary material for: Loss of cardiolipin and porins bypasses the essentiality of the sigma E cell envelope stress response in Escherichia coli
Source: mBio. 2025 Aug 18;16(9):e01613-25. doi: 10.1128/mbio.01613-25 (PMC12421810; doi:10.1128/mbio.01613-25)
Supplement: Supplemental Figures — Figures S1 to S5. [file mbio.01613-25-s0001.docx]

**Loss of cardiolipin and porins bypasses the essentiality of the sigma E cell envelope stress response in *Escherichia coli***

Zihao Yang^1,2^, Emily C.A. Goodall^1,2^, Bing Zhang^1,2^, Von V.L. Torres^1,2^, Jessica L. Rooke^1,2^, Karthik Pullela^1,2^, Rochelle M. Da Costa^1,2^, Chris Icke^1,2^, Mark Blaskovich^1,2^, Simon Legood^3^, Adam F Cunningham^3^, Waldemar Vollmer^1,2^, Jack A. Bryant^4^, Ian R. Henderson^1,2^

^1^Institute for Molecular Bioscience, and the ^2^Centre for Superbug Solutions, University of Queensland, Brisbane, 4072, Australia

^3^Institute of Microbiology and Infection, University of Birmingham, United Kingdom

^4^School of Life Sciences, University of Nottingham, United Kingdom

*Corresponding author: i.henderson@uq.edu.au

Key words:

*E. coli* / cardiolipin / phospholipid / transposon sequencing / tradis


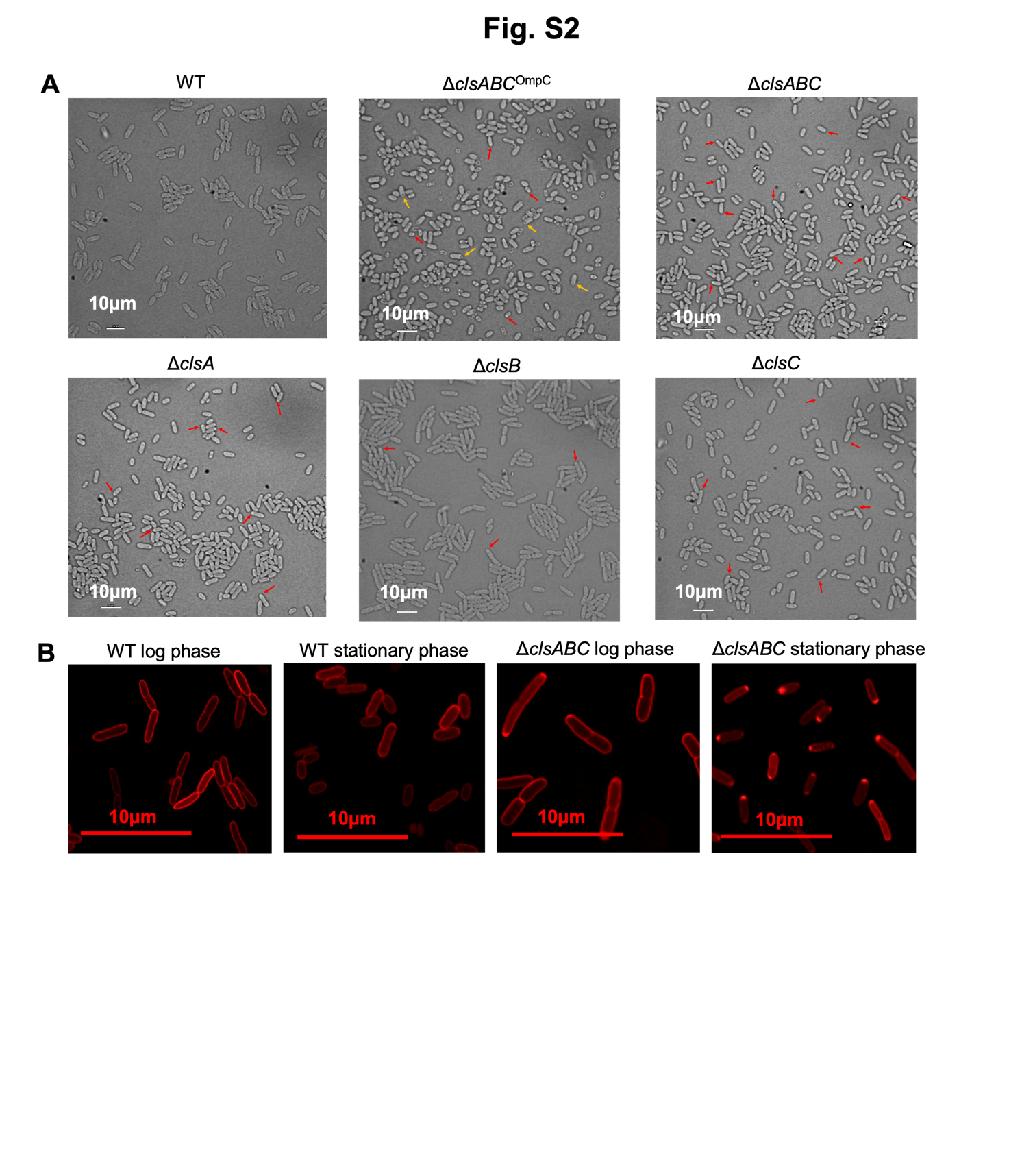


**Fig. S1. Cell morphology of *E. coli* BW25113 and *cls* mutants under** **DIC (A) and confocal microscopy (B)*.*** Representative DIC microscopy images of *E. coli* BW25113, *E. coli* BW25113 Δ*clsABC*^OmpC^, *E. coli* BW25113 Δ*clsABC*, BW25113 Δ*clsA*, BW25113 Δ*clsB*, and BW25113 Δ*clsC* at stationary phase; The representative confocal microscopy images of *E. coli* BW25113 and *E. coli* BW25113 Δ*clsABC* harbouring pASK pelB mcherry at log phase and stationary phase.


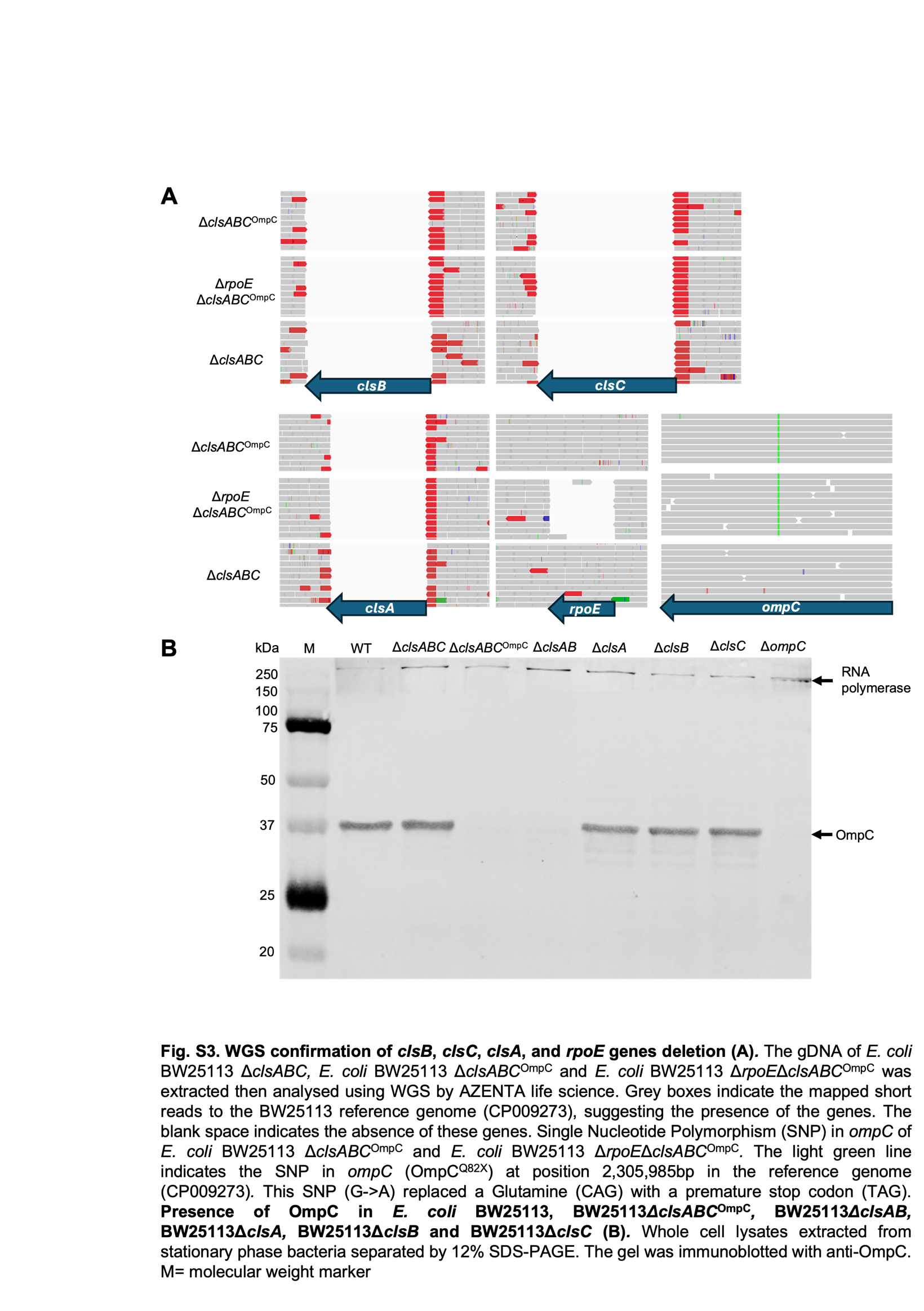


**Fig. S2. Confirmation of gene deletion mutants.** (A) The gDNA of *E. coli* BW25113 Δ*clsABC, E. coli* BW25113 Δ*clsABC*^OmpC^ and *E. coli* BW25113 Δ*rpoE*Δ*clsABC*^OmpC^ was extracted then analysed using WGS by AZENTA life science. Grey boxes indicate the mapped short reads to the BW25113 reference genome (CP009273), suggesting the presence of the genes. The blank space indicates the absence of these genes. The light green line indicates the SNP in *ompC* (OmpC^Q82X^) at position 2,305,985bp in the reference genome (CP009273)*.* This SNP (G->A) replaced a Glutamine (CAG) with a premature stop codon (TAG). (B) Presence of OmpC in *E. coli* BW25113, BW25113Δ*clsABC*^OmpC^*,* BW25113Δ*clsAB,* BW25113Δ*clsA,* BW25113Δ*clsB* and BW25113Δ*clsC.* Whole cell lysates extracted from stationary phase bacteria separated by 12% SDS-PAGE. The gel was immunoblotted with anti-OmpC. M= molecular weight marker. The molecular weight of OmpC is approximately 40 kDa.


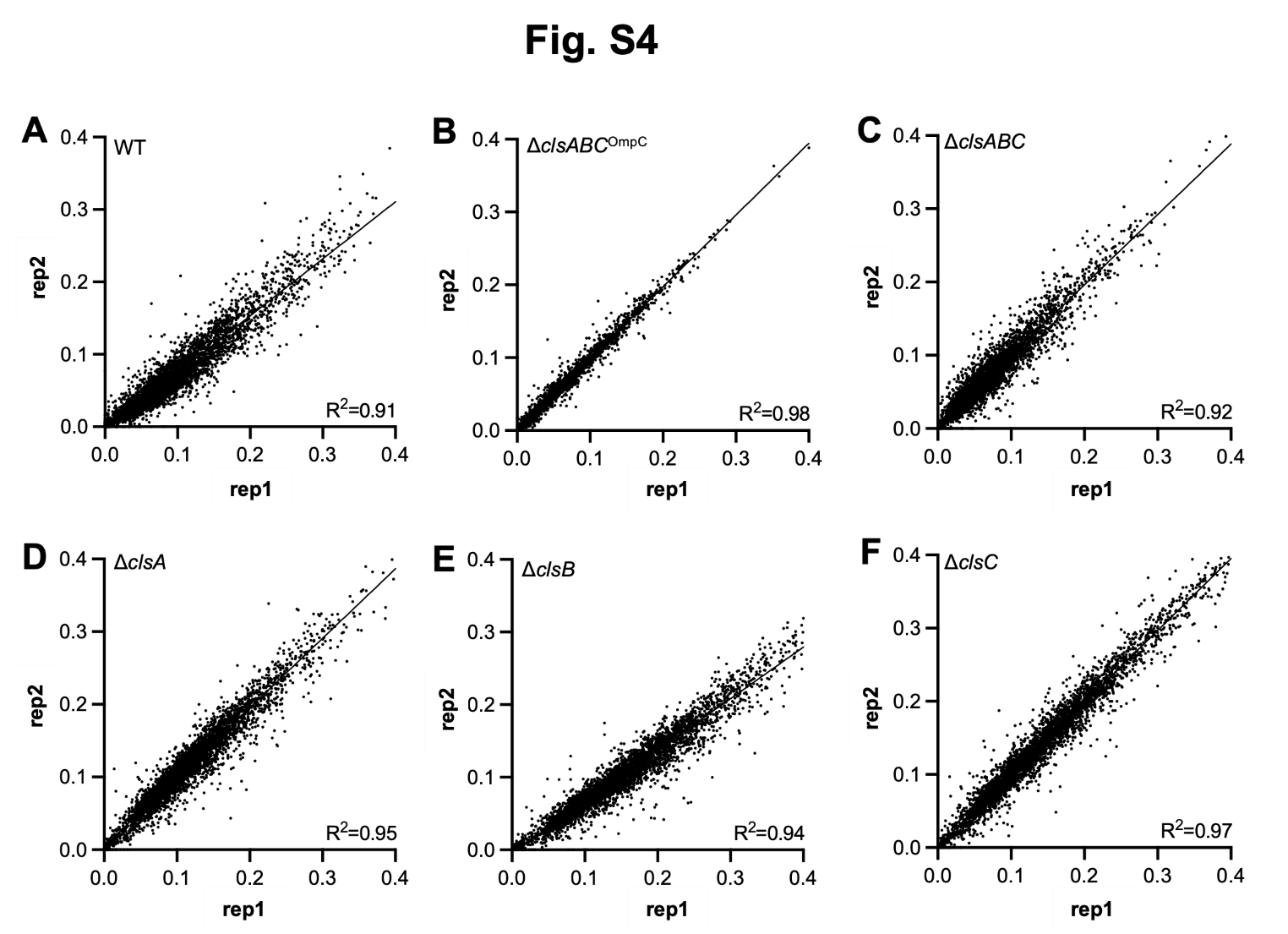


**Fig. S3.** Linear correlation of gene insertion index scores between sequencing replicates of TraDIS libraries in *E. coli* BW25113 (A), Δ*clsABC*^OmpC^ (B), Δ*clsABC* (C), Δ*clsA* (D), Δ*clsB* (E), and Δ*clsC* (F) deletion mutants.


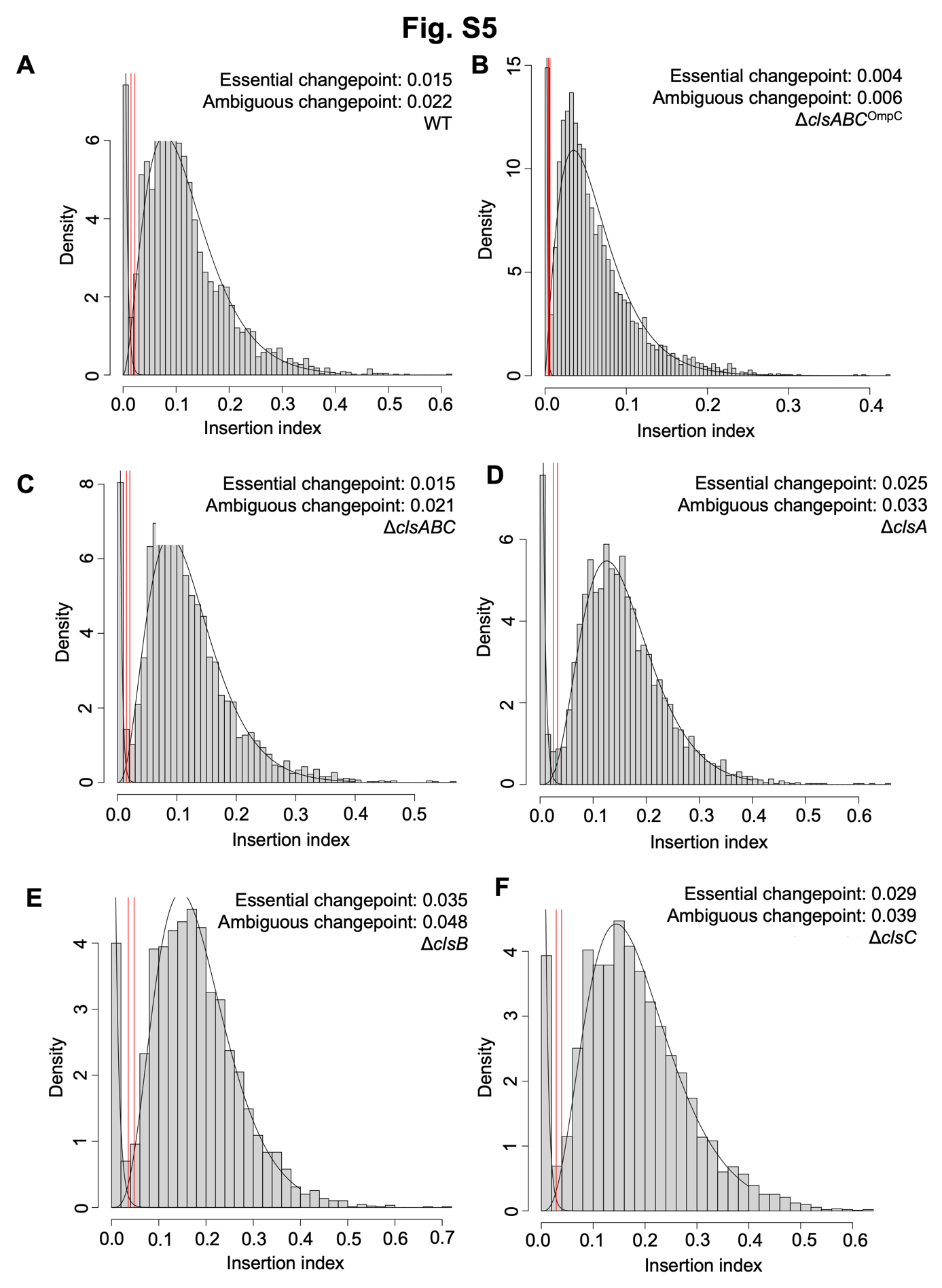


**Fig. S4. Bi-modal analysis.** The essentiality of each gene in *E. coli* BW25113 (A), Δ*clsABC*^OmpC^ (B), Δ*clsABC* (C), Δ*clsA* (D), Δ*clsB* (E), and Δ*clsC* (F) TraDIS libraries were identified using Bio-TraDIS bi-modal analysis toolkit. Description of the Bi-modal threshold: If the insertion index scores of genes are below the essential changepoint, the genes are identified as ‘essential’. If the insertion index scores of genes are higher than the ambiguous changepoint, the genes are identified as ‘non-essential’.


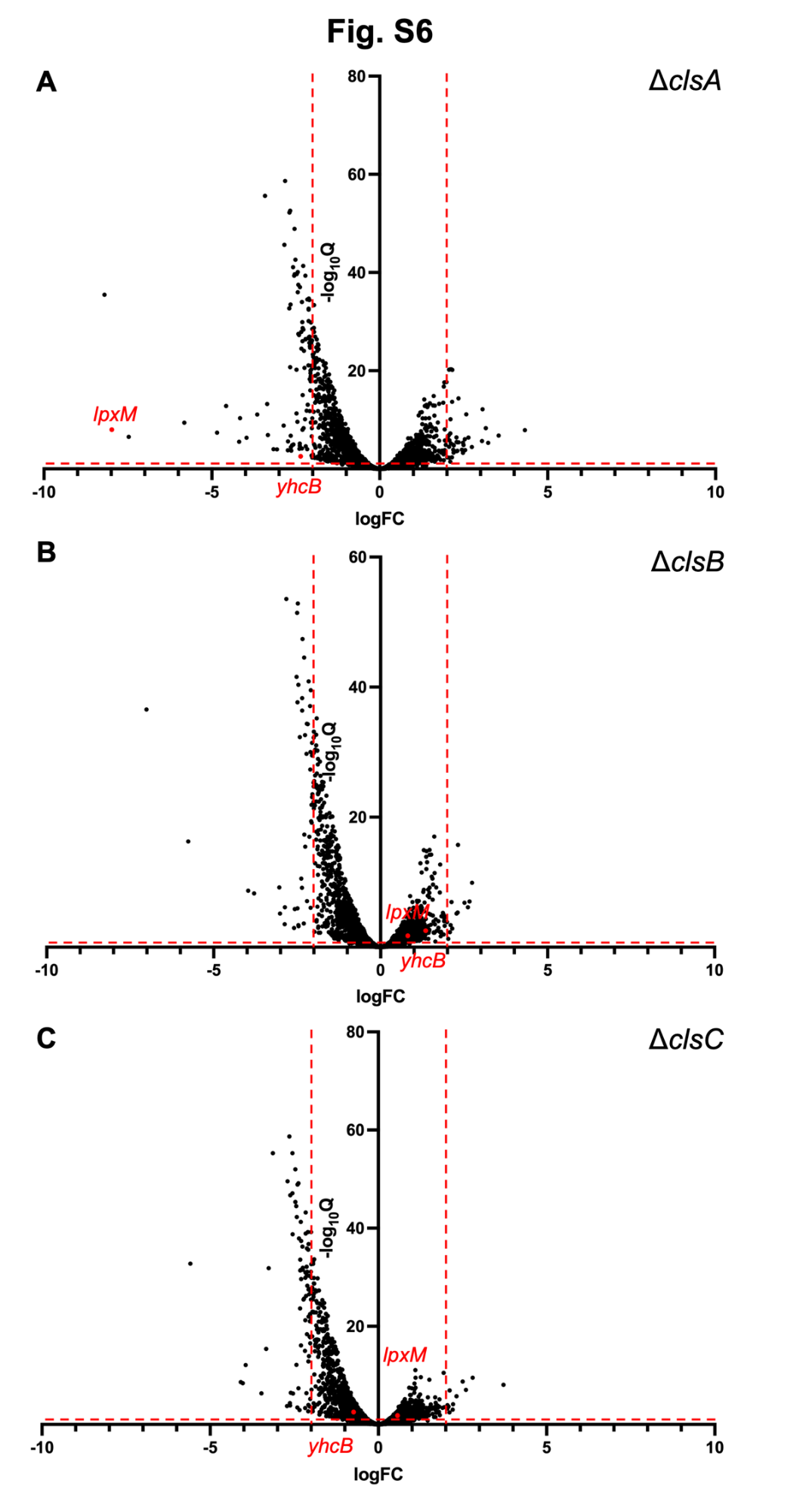


**Fig. S5. Volcano plots of gene fitness score of the TraDIS libraries** in *E. coli* BW25113 Δ*clsA* (A), Δ*clsB* (B), and Δ*clsC* (C) using the Bio-TraDIS toolkit. For each point, two-fold changes (log_2_FC) of normalised insertion sequencing read abundance per insertion site throughout the BW25113 genome were compared between each TraDIS library, with their respective adjusted p-values [q-value, represented here as -log_2_(q-value)].
