# Supplementary material for: Loss of cardiolipin and porins bypasses the essentiality of the sigma E cell envelope stress response in Escherichia coli
Source: mBio. 2025 Aug 18;16(9):e01613-25. doi: 10.1128/mbio.01613-25 (PMC12421810; doi:10.1128/mbio.01613-25)
Supplement: Tables S1 and S2 — Summary of and uniquely essential genes in TraDIS libraries. [file mbio.01613-25-s0002.docx]

**Table S1. Summary data of the TraDIS libraries.**

| Libraries | Total reads | Unique insertions sites | Insertion frequency ^a^ |
| --- | --- | --- | --- |
| WT BW25113 | 3,616,158 | 503,333 | 9.2 |
| Δ*clsABC*^OmpC^ | 2,708,478 | 262,866 | 17.6 |
| Δ*clsABC* | 2,215,502 | 500,060 | 9.3 |
| Δ*clsA* | 2,578,703 | 646,974 | 7.1 |
| Δ*clsB* | 3,066,222 | 755,284 | 6.1 |
| Δ*clsC* | 3,342,146 | 778,082 | 6.0 |

^a^ Insertion frequency = *E. coli* BW25113 genome length (4,631,469 bp) / unique insertion sites.

**Table S2. Genes in the TraDIS libraries deemed uniquely essential by the bi-modal analysis**

| Δ*clsABC*^OmpC^ (31) | Δ*clsABC* (59) | | Δ*clsA* (71) | | | Δ*clsB* (66) | | | Δ*clsC* (35) | |
| --- | --- | --- | --- | --- | --- | --- | --- | --- | --- | --- |
| *argW* | *acnB^#^* | *thrU* | *appY^#^* | *rlmE^#^* | *yhaC^#^* | *argV* | *rdlB* | *ykgR*^Δ^ | *argV* | *yffS** |
| *dnaQ*^Δ^ | *appY^#^* | *thyA** | *argV* | *rpiA^#^* | *yhcB*^Δ^ | *asnU* | *rlmE^#^* | *ymfD^#^* | *csrA** | *ygeF** |
| *folB** | *cmk^#^* | *tilS** | *ariR^#^* | *rsgA** | *yjbL^#^* | *cmk^#^* | *rpmE^#^* | *ymfE** | *cysE*^Δ^ | *ymfD^#^* |
| *folC** | *cspI^#^* | *ubiD** | *cmk^#^* | *secB^#^* | *ykgR*^Δ^ | *cysE*^Δ^ | *rsgA** | *ynfN^#^* | *folB** | *ymfE** |
| *glnU* | *csrA** | *uof*^Δ^ | *cspI^#^* | *secD^#^* | *ymfD^#^* | *dsrA* | *rttR* | *ypjC^#^* | *folC** | *ypjC^#^* |
| *glyA** | *dcd** | *valY* | *csrA** | *secF^#^* | *ymfE** | *elaD^#^* | *secB^#^* | *zwf^#^* | *folP** |  |
| *gnsB^#^* | *elaD^#^* | *ybfB^#^* | *cysE*^Δ^ | *tadA** | *ymjC*^Δ^ | *fdx** | *secD^#^* |  | *glnU* |  |
| *gpsA** | *fdx** | *yccE^#^* | *elaD^#^* | *tdcR*^Δ^ | *ynfN^#^* | *folB** | *tadA** |  | *glnW* |  |
| *ldrA*^Δ^ | *folB** | *yciE*^Δ^ | *folB** | *thrU* | *ypjC^#^* | *folC** | *thrU* |  | *glyA** |  |
| *lpxM^#^* | *folC** | *yddL** | *folC** | *thyA** | *yqeK^#^* | *folK^#^* | *thyA** |  | *gpsA** |  |
| *metT* | *folP** | *ydeO*^Δ^ | *folP** | *tilS** | *yqeL** | *folP** | *tilS** |  | *iraM** |  |
| *phoU^#^* | *glyA** | *ydgU*^Δ^ | *glnU* | *tpr^#^* |  | *glnU* | *tpr^#^* |  | *leuU* |  |
| *ppiB^#^* | *gnsB^#^* | *yedN** | *glnW* | *tusA^#^* |  | *glnW* | *tusB*^Δ^ |  | *lipB*^Δ^ |  |
| *psaA* | *holC^#^* | *yehC^#^* | *glyA** | *ubiD** |  | *glyA** | *ubiD** |  | *metT* |  |
| *rdlC* | *hold** | *yehD*^Δ^ | *glyW* | *ubiF^#^* |  | *glyW* | *ubiH** |  | *nusA** |  |
| *rspR^#^* | *hscA** | *yfdF^#^* | *gpsA** | *ubiH** |  | *gnsB^#^* | *ybfB^#^* |  | *pheU* |  |
| *rydB* | *hscB** | *yffS** | *higA** | *ybfB^#^* |  | *gpsA** | *yciE*^Δ^ |  | *phoU^#^* |  |
| *rydC* | *ileX* | *yfjW^#^* | *hold** | *yccE^#^* |  | *ileX* | *yciU^#^* |  | *ppiB^#^* |  |
| *secB^#^* | *iraM** | *ygeF** | *ileX* | *yddL** |  | *iraM** | *yddL** |  | *psd** |  |
| *tadA** | *kilR^#^* | *ygeI*^Δ^ | *iraM** | *ydgU*^Δ^ |  | *leuU* | *ydgU*^Δ^ |  | *ptsH*^Δ^ |  |
| *thyA** | *leuU* | *ygeK^#^* | *kilR^#^* | *yedN** |  | *lipB*^Δ^ | *yedN** |  | *rcbA*^Δ^ |  |
| *tilS** | *lpxM^#^* | *yjbL^#^* | *lipB*^Δ^ | *yehC^#^* |  | *lysV* | *yehC^#^* |  | *rlmE^#^* |  |
| *ydgU*^Δ^ | *pheL^#^* | *yjbS** | *lpxM^#^* | *yehD*^Δ^ |  | *metT* | *yffS** |  | *secD^#^* |  |
| *yecJ^#^* | *pheU* | *ymfD^#^* | *metT* | *yfjW^#^* |  | *pheU* | *yfjW^#^* |  | *tadA** |  |
| *yeeH*^Δ^ | *ppiB^#^* | *ymfE** | *pheU* | *ygeF** |  | *phoU^#^* | *ygeF** |  | *thrU* |  |
| *yffS** | *psd** | *yoaI*^Δ^ | *phoU^#^* | *ygeG** |  | *ppiB^#^* | *ygeI*^Δ^ |  | *thyA** |  |
| *yhcB*^Δ^ | *rcbA*^Δ^ | *ypjC^#^* | *ppiB^#^* | *ygeI*^Δ^ |  | *psd** | *ygeK^#^* |  | *tilS** |  |
| *yjeV*^Δ^ | *rsgA** | *yqeJ^#^* | *psd** | *ygeK^#^* |  | *ptsH*^Δ^ | *ygfZ** |  | *ubiD** |  |
| *ykfN*^Δ^ | *secD^#^* | *yqeK^#^* | *ptsI** | *ygeN** |  | *ptsI** | *yjbL^#^* |  | *ubiH** |  |
| *ymfD^#^* | *secF^#^* |  | *rcbA*^Δ^ | *ygfZ** |  | *rcbA*^Δ^ | *yjbS** |  | *ybfB^#^* |  |
| *yoaI*^Δ^ |  |  |  |  |  |  |  |  |  |  |

* Genes that are reported essential in prior studies

^Δ^ Genes that are reported non-essential in prior studies

^#^ Genes that are reported unclear in prior studies

() number of the genes
