# Supplementary material for: Loss of cardiolipin and porins bypasses the essentiality of the sigma E cell envelope stress response in Escherichia coli
Source: mBio. 2025 Aug 18;16(9):e01613-25. doi: 10.1128/mbio.01613-25 (PMC12421810; doi:10.1128/mbio.01613-25)
Supplement: Tables S5 to S8 — Strains, plasmids, primers, and antibodies. [file mbio.01613-25-s0003.docx]

**Table S5. Bacterial strains used in the study.**

| Bacterial strain | Description | Reference |
| --- | --- | --- |
| *E. coli* BW25113 | *E. coli* K-12 derivative, *lacI^q^ rrnB*_T14_ Δ*lacZ*_WJ16_ *hsdR514* Δ*araBAD*_AH33_ Δ*rhaBAD*_LD78_ | [49] |
| *E. coli* DH5α | High efficiency competent *E. coli* cloning strain, *fhuA*2 (*argF*-*lacZ*) U169 *phoA glnV*44 80 (*lacZ*) M15 *gyrA*96 *recA*1 *relA*1 *endA*1 *thi*-1 *hsdR*17 | New England Biolabs |
| *E. coli* BW25113 ΔclsABC^OmpC^ | Δ*clsA*::*frt* Δ*clsB*::*frt* Δ*clsC*::*frt* OmpC^Q82X^ | [50] |
| *E. coli* BW25113 Δ*clsA* | Δ*clsA*::*frt* |  |
| *E. coli* BW25113 Δ*clsB* | Δ*clsB*::*frt* |  |
| *E. coli* BW25113 Δ*clsC* | Δ*clsC*::*frt* |  |
| *E. coli* BW25113 *ΔtolC* | Δ*tolC*::*frt* | This study |
| *E. coli* BW25113 *ΔtolC* ΔclsABC^OmpC^ | Δ*clsA*::*frt* Δ*clsB*::*frt* Δ*clsC*::*frt* Δ*tolC*::*frt* OmpC^Q82X^ |  |
| *E. coli* BW25113 Δ*clsABC* Δ*rpoE* OmpC^Q82X^ | Δ*clsA*::*frt* Δ*clsB*::*frt* Δ*clsC*::*frt* Δ*rpoE*::*frt* OmpC^Q82s^ |  |
| *E. coli* BW25113 Δ*clsABC* | Δ*clsA*::*frt* Δ*clsB*::*frt* Δ*clsC*::*frt* |  |

**Table S6. Plasmids used in the study.**

| Plasmid | Description | Reference |
| --- | --- | --- |
| pDOC-K | pDOC plasmid with kanamycin resistance cassette flanked by fragment length polymorphism (FLP) sites, Kan^R^. | [51] |
| pKD4 | Contains kanamycin resistance cassette flanked by FLP sites, Kan^R^. | [42] |
| pKD46 | Heat sensitive plasmid containing a L-arabinose inducible promoter for λ Red recombinase expression, Amp^R^. | [42] |
| pACBSR | Contains a L-arabinose inducible promoter for λ Red recombinase expression, Cm^R^. | [52] |
| pCP20 | Heat sensitive plasmid for FLP recombinase expression and kanamycin cassette removal, Amp^R^. | [42] |
| pFLP-BSR | pACBSR carrying FLP recombinase for kanamycin cassette removal, Cm^R^. | [53] |
| pRseAB | IPTG inducible vector, *rseA* and *rseB* in pTrc99a, Carb^R^ | [29] |
| pTrc99a | IPTG inducible bacterial expression vector with lacI promoter, pBR322 ori Carb^R^ | [54] |
| pLUX | Luciferase vector, Kan^R^ | [55] |
| pLUX-P*rpoE* | *rpoE*-luciferase reporter, Kan^R^; *rpoE* promoter cloned between *Xho*I and *Bam*HI restriction sites of pLUX | This work |
| pASK-pelB-mcherry | pASK plasmid with *pelB* signal sequence tagged with mCherry | This work |
| pBAD/myc-  HisA | L-arabinose inducible promoter upstream of a C-terminal myc tag and poly-His tag, Carb^R^. | Invitrogen |

**Table S7. Primers used in the study.**

| **Primer** | **Sequence (5’-3’)** | **Description** |
| --- | --- | --- |
| *clsA*_check_F | CCTCAAGCCAACGCGATTTACG | Forward primer to check the size of *clsA* in BW25113 |
| *clsA*_check_R | CCAGACCAATCACCACTTCG | Reverse primer to check the size of *clsA* in BW25113 |
| *clsB*_KO_F | TGGCGACACCTTTCTGATCATGCCCCTTTAAGTGCGGAGATTCATTTATGGTGTAGGCTGGAGCTGCTTC | Forward primer to construct Δ*clsB*::*aph* in BW25113 |
| *clsB*_KO_R | TAAGCGCCAGCGCGGGTGTGATTTACTCATCAGGGTTTTACCCCCGTGTTCATATGAATATCCTCCTTA | Reverse primer to construct Δ*clsB*::*aph* in BW25113 |
| *clsB*_check_F | CGATGTTTCGGTCGATGGTGC | Forward primer to check the size of *clsB* in BW25113 |
| *clsB*_check_R | CGATTGTAGTCGCGGATGACC | Reverse primer to check the size of *clsB* in BW25113 |
| *clsC*_KO_F | CGCCCACCTCTACGAAAGACTCCTTACCCAACAAGGAGATGAATGATTTGGTGTAGGCTGGAGCTGCTTC | Forward primer to construct Δ*clsC*::*aph* in BW25113 |
| *clsC*_KO_R | CGATAAAGCACCAGCCCGTTAAGCCACATTTACAATAACCATTCCACGGGCATATGAATATCCTCCTTA | Reverse primer to construct Δ*clsC*::*aph* in BW25113 |
| *clsC*_check_F | GCAGGATGCCTATCTCAATAGC | Forward primer to check the size of *clsC* in BW25113 |
| *clsC*_check_R | GCATCGCTGTTTATCTATCTGG | Reverse primer to check the size of *clsC* in BW25113 |
| *rpoE*_KO_F | CGTTTCGATAGCGCGTGGAAATTTGGTTTGGGGAGACTTTACCTCGGATGGTGTAGGCTGGAGCTGCTTC | Forward primer to construct Δ*rpoE*::*aph* in BW25113 |
| *rpoE*_KO_R | TAATACCCTTATCCAGTATCCCGCTATCGTCAACGCCTGATAAGCGGTTGCATATGAATATCCTCCTTA | Reverse primer to construct Δ*rpoE*::*aph* in BW25113 |
| *rpoE*_check_F | GCTCAAATTGCAGCTAATGG | Forward primer to check the size of *rpoE* in BW25113 |
| *rpoE*_check_R | CTTATCCAGTATCCCGCTATC | Reverse primer to check the size of *rpoE* in BW25113 |

**Table S8. Antibodies used in the study.**

| Antibody | Type | Animal | Concentration | Reference |
| --- | --- | --- | --- | --- |
| IRDye^®^ 800CW Goat anti-Rabbit IgG | Secondary | Goat | 1: 15,000 | Li-Cor or Abcam |
| IRDye^®^ 680LT Goat anti-Mouse IgG | Secondary | Goat | 1: 15,000 | Li-Cor or Abcam |
| α-*E. coli* RNA Polymerase β | Primary | Mouse | 1: 10,000 | BioLegend |
| Anti-OmpC | Primary | Rabbit | 1: 5,000 | Sigma-Aldrich |
| Anti-OmpF | Primary | Rabbit | 1: 5,000 | Sigma-Aldrich |
